# Supplementary material for: WIPI2 Links LC3 Conjugation with PI3P, Autophagosome Formation, and Pathogen Clearance by Recruiting Atg12–5-16L1
Source: Mol Cell. 2014 Jul 17;55(2):238–52. doi: 10.1016/j.molcel.2014.05.021 (PMC4104028; doi:10.1016/j.molcel.2014.05.021)
Supplement: Document S1. Supplemental Experimental Procedures, a list of primers used in this study, and Figures S1–S7 [file mmc1.pdf]

Molecular Cell, Volume *55*

Supplemental Information

WIPI2 Links LC3 Conjugation with PI3P, Autophagosome Formation, and Pathogen Clearance by Recruiting Atg12–5-16L1

Hannah C. Dooley, Minoo Razi, Hannah E.J. Polson, Stephen E. Girardin, Michael I. Wilson, and Sharon A. Tooze

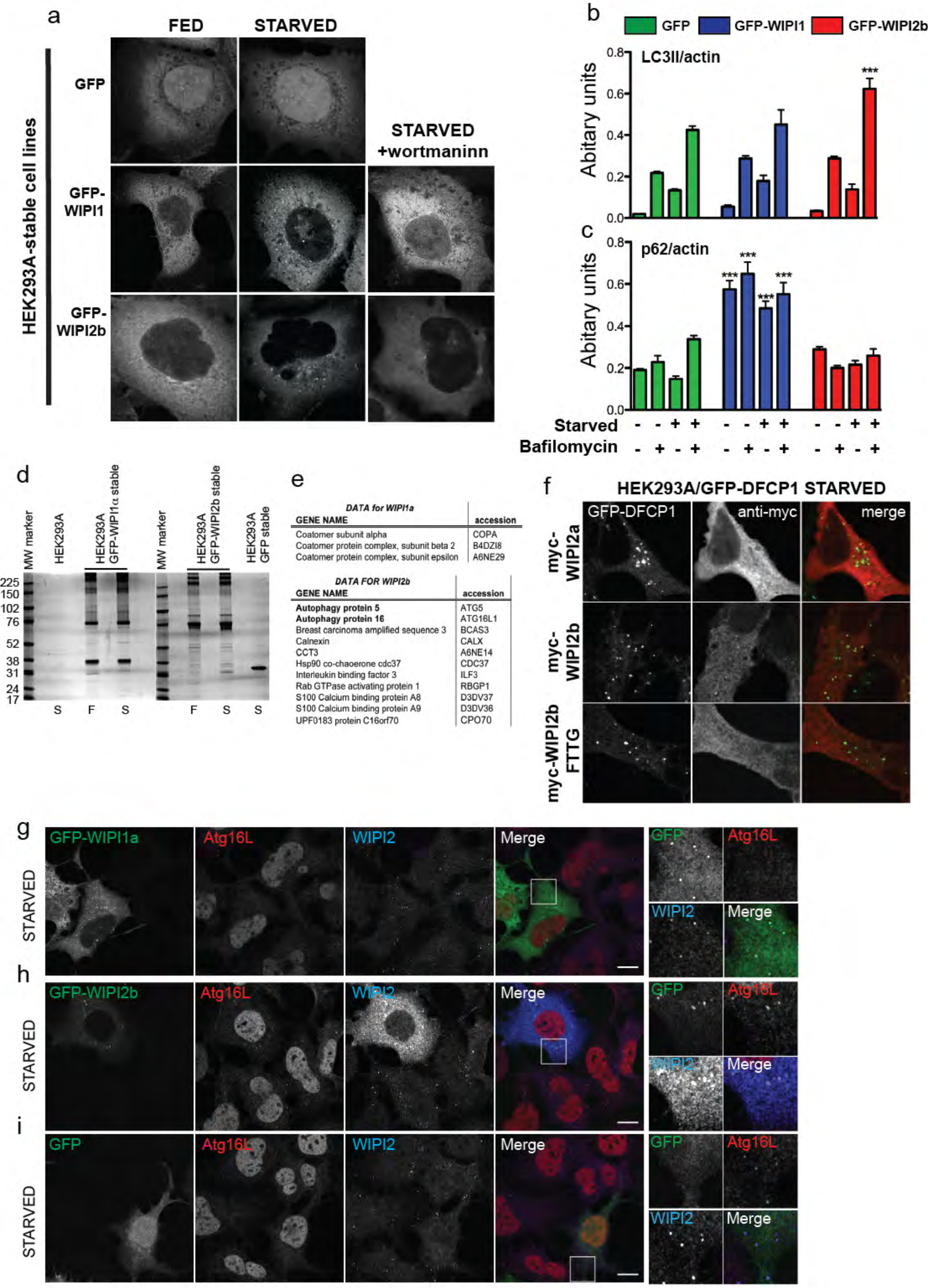

Figure S1, related to Figure 1

Figure S1, related to Figure 1.

Characterization of GFP, GFP-WIPI1 and WIPI2b stable cell lines and GFP-TRAP pulldown. ***(a)*** HEK293A stable cell lines were incubated in full medium (fed), EBSS (starved) or EBSS plus wortmannin (Starved + wortmannin) for 2 hrs then fixed and visualized by confocal microscopy. ***(b, c)*** Analysis of flux in stable cell lines. GFP, GFP-WIPI1 or GFP-WIPI2b cells (in triplicates) were incubated with full medium (Fed), full medium with BafA (Fed+Baf), EBSS (Starved) and EBSS with BafA (Starved+BafA) for 2 hrs. LC3-II and p62 signals were quantified and normalized to actin. Error SEM for three independent experiments is shown. Statistical analysis was performed by one-way anova. ***, $p<0.0005$. ***(d)*** Protein complexes were purified from stable cell lines (GFP, GFP-WIPI1 or GFP-WIPI2b) from both fed and starved conditions using GFP-Trap® subjected to SDS-PAGE and stained with colloidal stain. ***(e)*** A list of identified interacting proteins after comparison and removal of those pulled down from cells expressing GFP alone or untransfected HEK293 cells. ***(f)*** WIPI2b colocalises with DFCP-1 but WIPI2a does not. Myc-tagged WIPI2a, WIPI2b and WIPI2b FTTG were transfected into GFP-DFCP1 stable cell lines. After 24 hrs the cells were starved in EBSS for 2 hrs, fixed and labelled with anti-myc antibody. GFP-DFCP1 and myc-tagged proteins were then visualized by confocal microscopy. ***(g-i)*** Cells stably expressing (g) GFP-WIPI1a, (h) GFP-WIPI2b, or (i) GFP were starved for 2 hrs in EBSS before visualisation by confocal microscopy using antibodies against endogenous Atg16L1 and WIPI2. Scale bars are equal to 10 μm. Note: In (g) in the untransfected cell (bottom right) endogenous WIPI2 colocalizes with Atg16L1.

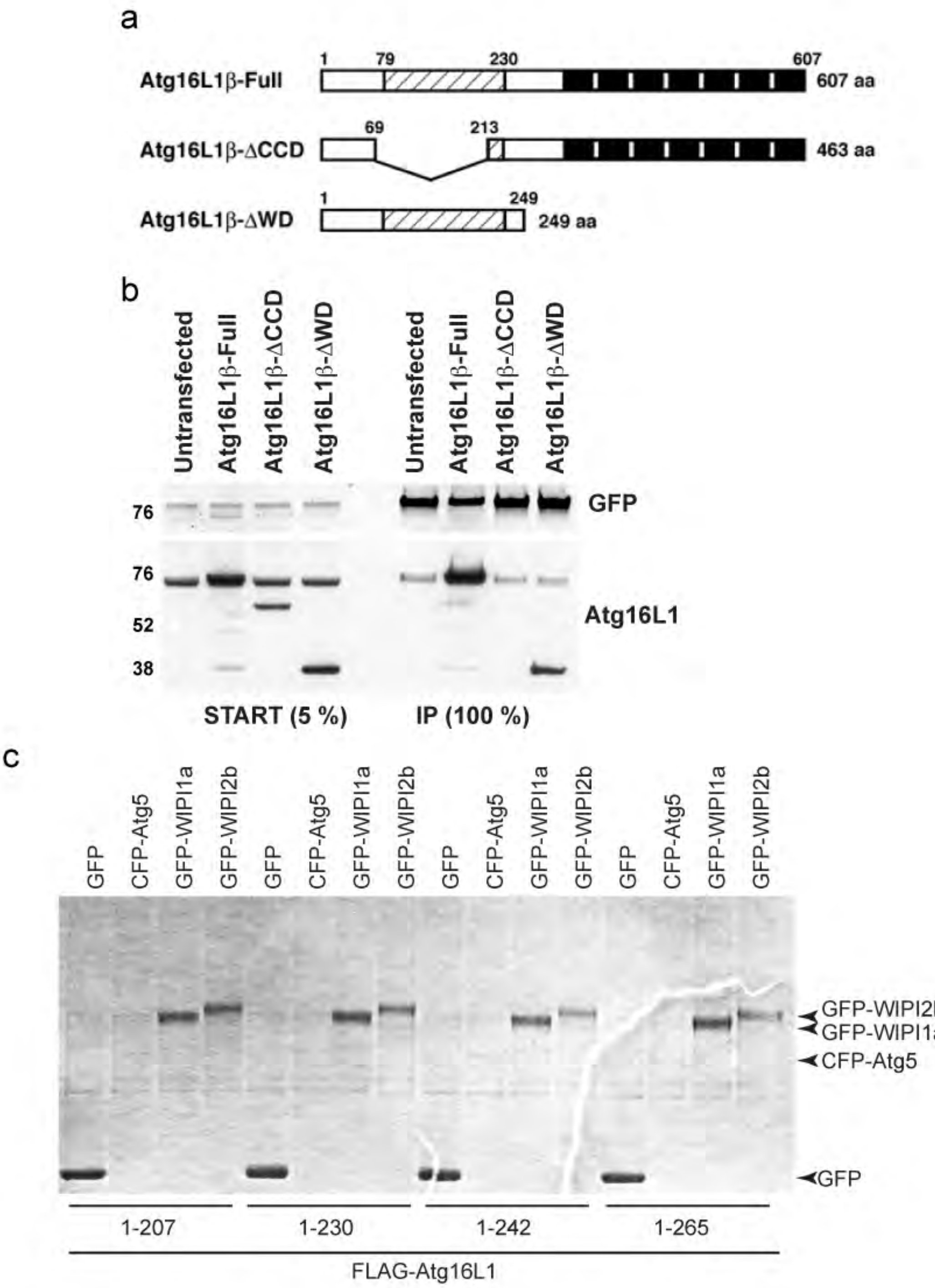

Figure S2, related to Figure 2

Figure S2, related to Figure 2.

Mapping interaction with Atg16L1. ***(a and b)*** Residues 1-249 and the coil-coil domain of human Atg16L1 are required for interaction with WIPI2b. Human Atg16L1b constructs were transfected into HEK293A cells stably expressing GFP-WIPI2b and isolated using GFP-TRAP® followed by immunoblotting with anti-Atg16 antibody. ***(c)*** Commassie blue stained gel of GFP-Trap® samples from Fig. 2c.

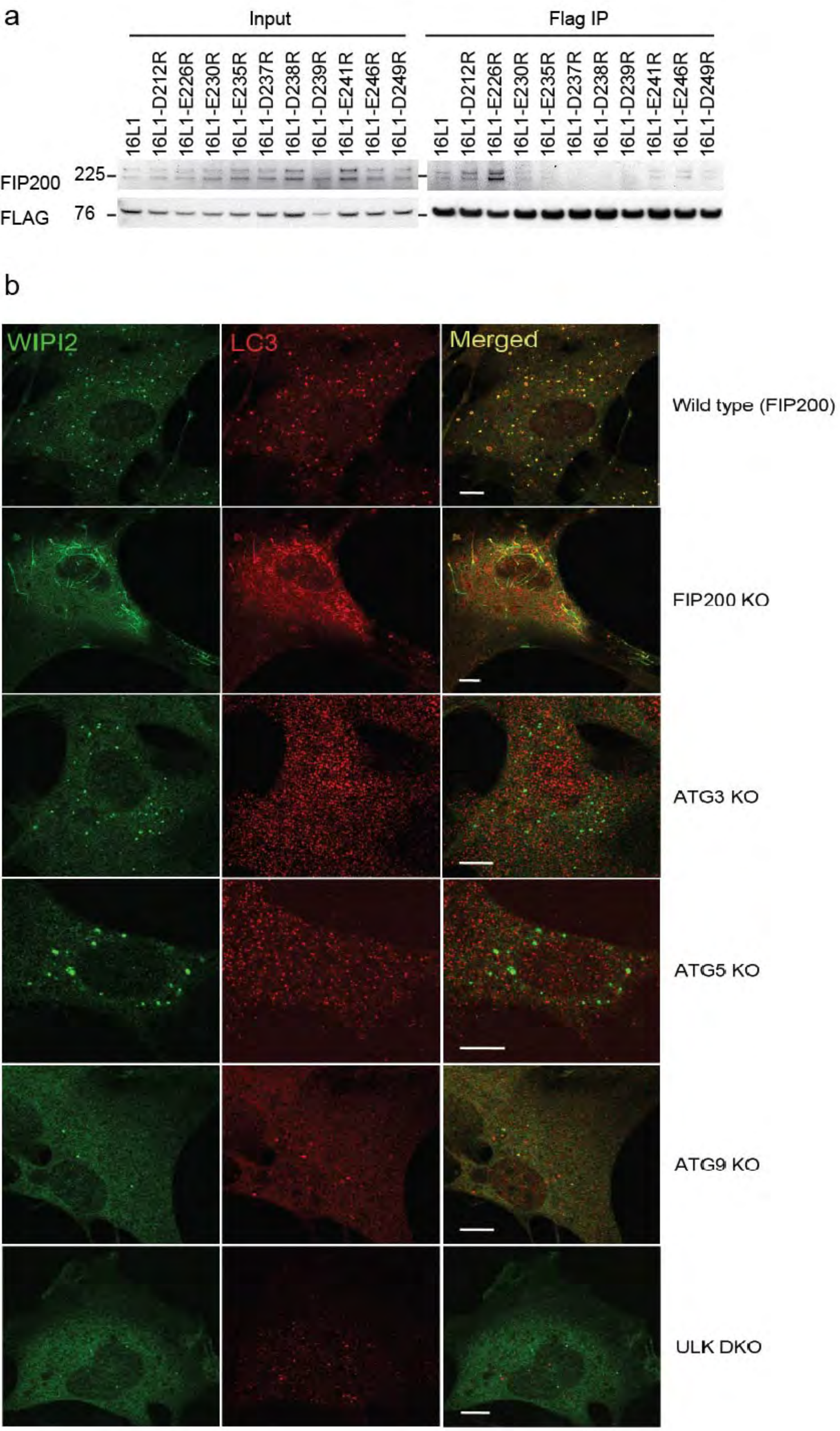

Figure S3, related to Figure 3

Figure S3, related to Figure 3. Mapping the Atg16L1 binding site in FIP200, and the localization of WIPI2 in FIP200 and other Atg-mutant MEFS. ***(a)*** Cell lysates from HEK293A cells transiently expressing GFP-WIPI2b were mixed with lysates from HEK293A cells transiently expressing FLAG-Atg16L1, or the FLAG-Atg16L1 mutants shown, and immunoprecipitated using FLAG M2 agarose beads. Tagged-protein input and bound were analysed by immunoblotting using anti-FIP200 and anti-FLAG antibodies. ***(b)*** Endogenous WIPI2 localization in FIP200, Atg3, Atg5, Atg9 and ULK1/2 Knockout MEFs. Cells were starved in EBSS for 90 mins and then fixed. WIPI2 was labelled with a monoclonal anti-WIPI2 (2A2) while LC3 was labelled with a rabbit anti-LC3 (Abcam). WIPI2 forms puncta in wild-type cells as expected. In 10% of FIP200 KO MEFs, WIPI2 is present on long tubules decorated with LC3, while the remaining 90% have undetectable WIPI2 or LC3. In Atg3 and Atg5 KO MEFS WIPI2 forms puncta that are not LC3 positive. Atg9 and ULK DKO are as previously described (McAlpine et al., 2013, Orsi et al., 2012). Atg9 KO MEFS has a small number of WIPI2-LC3-positive puncta while ULK DKO has fewer, which are not LC3-positive. Scale bars are 10μm.

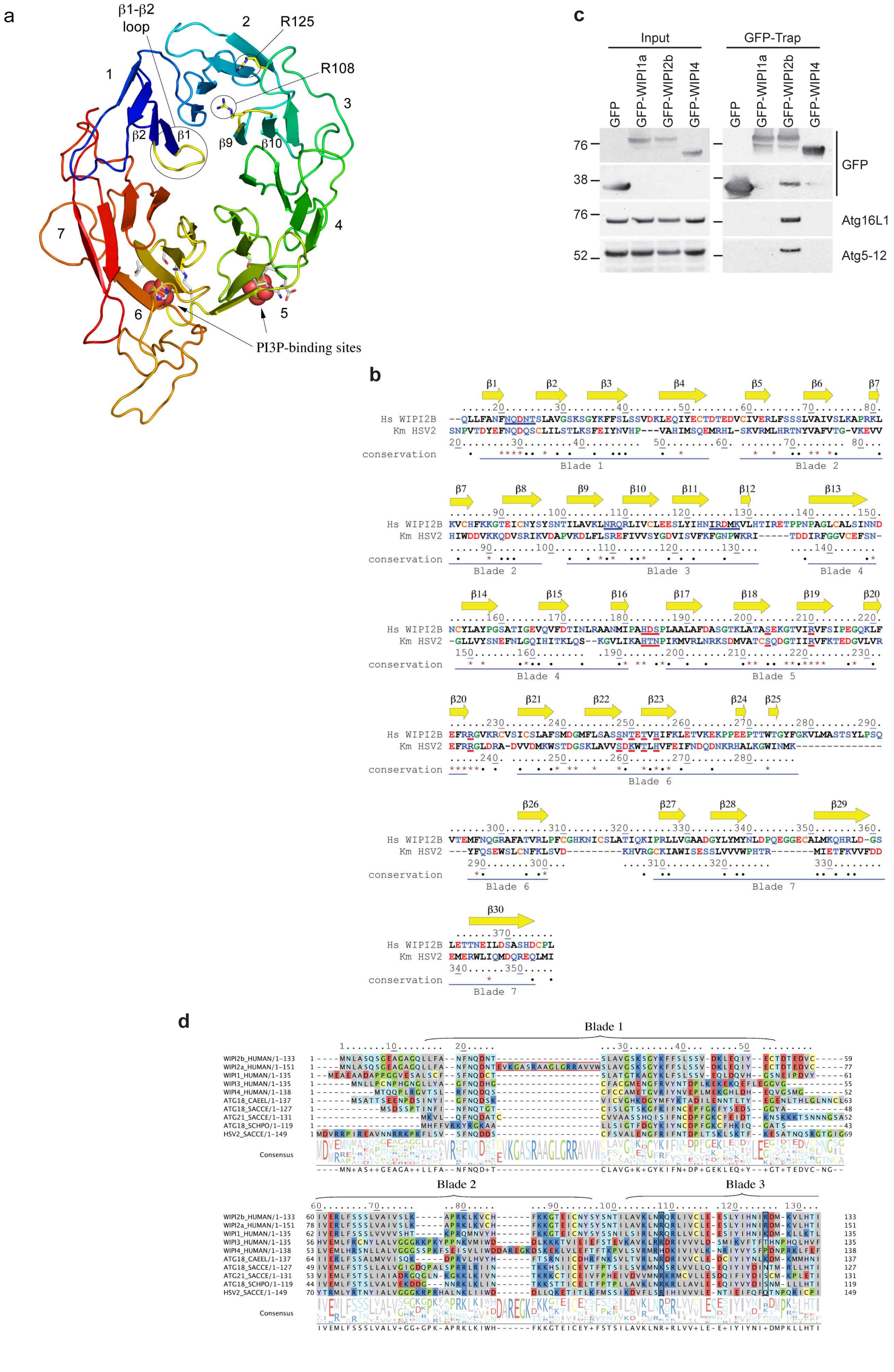

Figure S4, related to Figure 4

Figure S4, related to Figure 4. ***(a)*** A model for human WIPI2b (14-377) based on the X-ray structure of *Kluyvelomyces marxianus* Hsv2 homologue (Watanabe et al., 2012);3vu4.pdb) made with the iterative threading assembly refinement (I-TAASER;(Roy et al., 2010)). The cartoon is colored from the N-terminus with blue to red gradient. The b1-b2 and b9-b10 loops are colored yellow, R108 and R125 are shown as sticks (highlighted in the alignment with "_"). Residues that bind PI3P (highlighted in the alignment with "_") and sulfates bound in 3vu4.pdb are shown as sticks and sphere representations, respectively. ***(b)*** Structural alignment of the WIPI2b model and HSV2 (3vu4.pbd). Secondary structure in the is from 3vu4.pdb and numbering in the alignment is from Uniprot Q9Y4P8 for WIPI2 isoform 4 (WIPI2b), and J3QW34 for *Kluyvelomyces marxianus* Hsv2. Conservation symbols are '*'=identity and '•'=functional equivalence. The WIPI2b model aligned to 316 of 319 residues in 3vu4:A with an RMSD <0.89. There were 34 additional residues in loops of WIPI2b model, with half these residues (17) inserted between b25-b26 of Blade 6. ***(c)*** WIPI4 does not interact with Atg16L1. GFP-WIPI4 was pulled down from HEK293 cells transfected with GFP-WIPI2 and GFP-WIPI4 and Atg16L1 was detected by immunoblotting using anti-Atg16 and anti-GFP antibodies. ***(d)*** Comparison of WIPI2b sequence to homologues. Alignment of following sequences: human WIPI1a (Q5MNZ9 isoform 1), human WIPI2a (Q9Y4P8 isoform 1), human WIPI2b (Q9Y4P8 isoform 4), human WIPI3 (Q5MNZ6), human WIPI4 (Q9Y484), *Saccharomyces cerevisiae* ATG18 (P43601), *Schizosaccharomyces pombe* ATG18a (Q9HDZ7), *Saccharomyces cerevisiae* ATG21 (Q02887) (insertion 53-98 not shown), *Saccharomyces cerevisiae* HSV2 (P50079), *Caenorhabditis elegans* ATG18a (O16466), was generated using Jalview (Waterhouse et al., 2009). Sequences and sequence numbering are from Uniprot (Consortium, 2013). Scale numbering is from WIPI2b. Normalized consensus sequence is shown below alignment. Residues are coloured by function and strength of colouring proportional to conservation above 15% identity in each column. The boundaries of Blade 1, 2 and 3 are indicated. The 18 aa insert in WIPI2a between β1-β2 is boxed in red. R108 and R125 positions are boxed in blue.

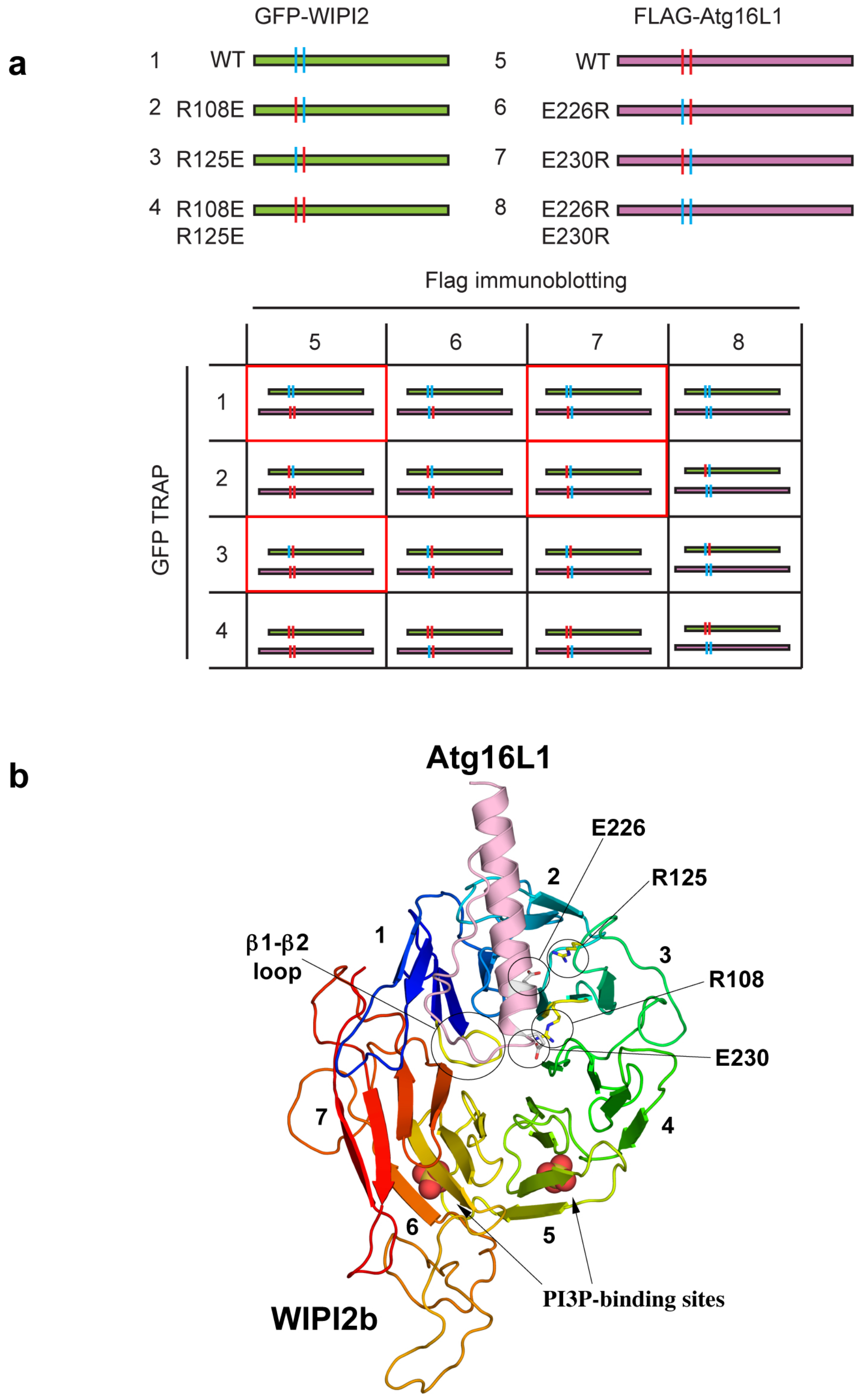

Figure S5, related to Figure 4

Figure S5, related to Figure 4. ***(a)*** **Charge-change experiment key for Figure 4d.** HEK293A cell lysates containing WIPI2b WT or point mutants no. 1-4 were mixed with HEK293A cell lysates expressing Atg16L1 WT or point mutants no. 5-8. In no. 1-4 blue vertical lines indicate the residues R108 and R125, while red vertical lines indicate the charge change mutation to E108 and E125. In no. 5-8 the red vertical lines indicate residues E226 and E230, while blue vertical lines indicate the mutation to R226 and R230. Following a GFP-TRAP® pulldown of WIPI2 the matrix indicates all the combinations tested. The boxes in red are those in which FLAG-Atg16L1 was detected after GFP-Trap® of WIPI2b (see Figure 4d). ***(b)*** **Model of Atg16L1 (207-246) (pink) docked onto WIPI2b (14-377) (rainbow)**. WIPI2b was modelled using 3vu4.pdb and Atg16L1 was modelled *ab initio* using I-TASSER (Roy et al., 2010). Both models were initially docked using ZDOCK for rigid-body protein-protein docking (Pierce et al., 2011), constrained by requiring E226 and E230 of Atg16L1, R108 and R125 of WIPI2b to be in the binding site. The top scoring docked model was then refined using RosettaDock (Lyskov and Gray, 2008) for local docking optimization. In the docking model shown, E230 interacts with R108.

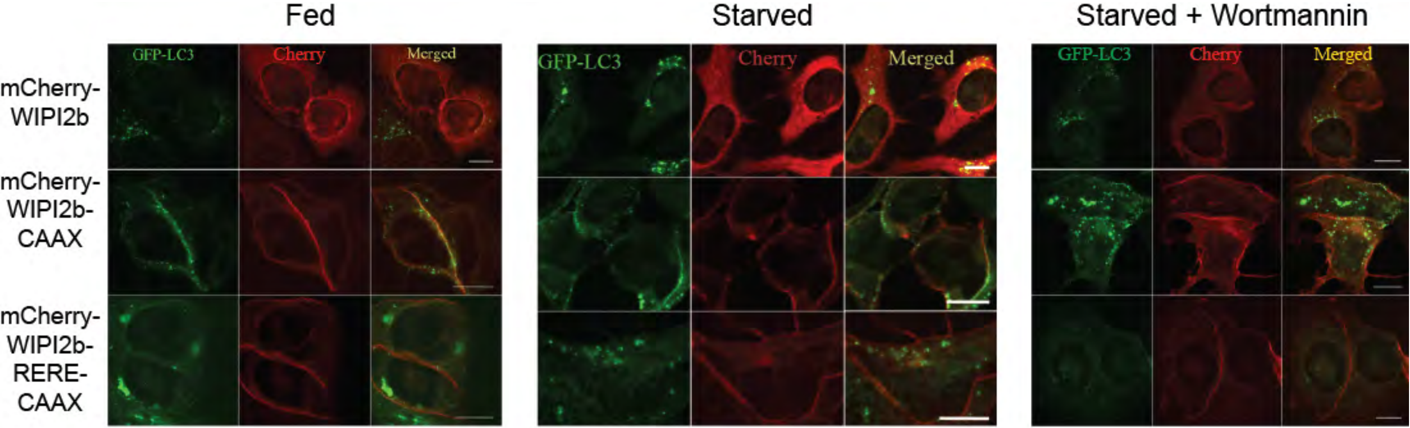

Figure S6, related to Figure 6

Figure S6, related to Figure 6. MCF7 WIPI2b-CAAX experiment. MCF7 cells transiently overexpressing the indicated mCherry constructs were either left in full medium (F), starvation medium (S) or starvation medium with wortmannin (W) for 2 hrs before being fixed and visualised by confocal microscopy. Scale bars are 10μm.

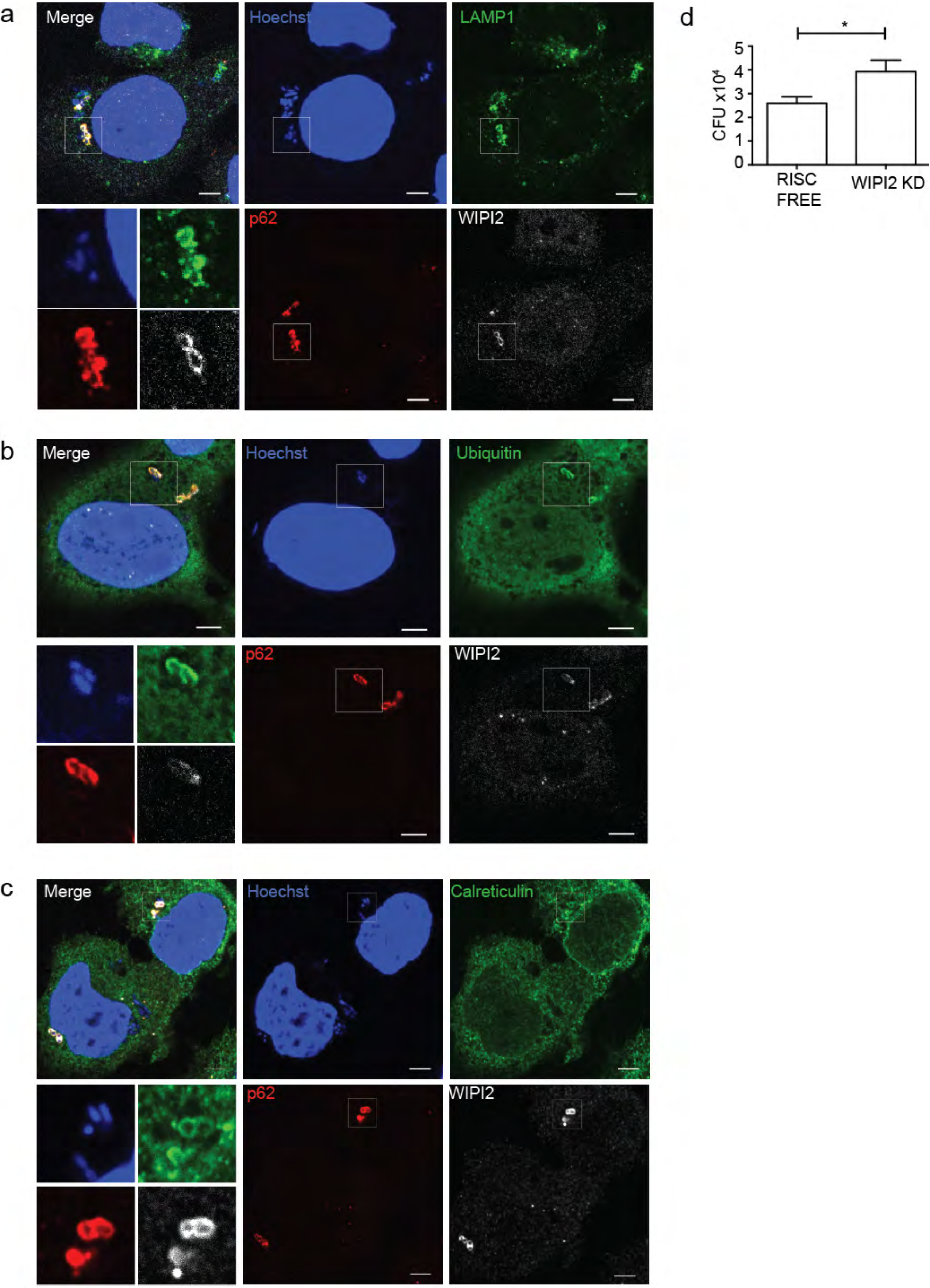

Figure S7, related to Figure 7

Figure S7, related to Figure 7. WIPI2-positive Salmonella are found in LAMP1 and UB-positive vesicles, which colocalize with ER marker calreticulin. ***(a, b, c)*** HeLa cells treated with RF siRNA for 72 hours were infected with Salmonella (MOI=100) for 1 hour. Cells were the fixed and labelled with the indicated antibodies for confocal microscopy analysis.

Loss of WIPI2 increases Salmonella proliferation. ***(d)*** HeLa cells treated with RF or WIPI2 siRNA for 72 hours were infected with Salmonella (MOI=100) for 1 hour and the colony-forming units (CFUs) determined. SEM for three independent experiments in triplicate is shown.

Movie S1, related to Figure 1. Live cell imaging of HEK293A cells stably expressing GFP-WIPI2b co-transfected with mCherry-Atg16L1. Cells were placed into EBSS at 37°C and immediately imaged using an UltraVIEW Perkin-Elmer spinning disk confocal microscope. Arrows mark the WIPI2b-positive structures shown in Fig. 1k. The arrows appear in the sequence shown in Fig. 1k, top, middle and bottom panels.

Extended Experimental Procedures:

Cell culture reagents

HeLa cells, HEK293A cells and their derivatives were grown in full medium: DMEM supplemented with 10% fetal calf serum and 4.8 mM L-glutamine. MEF cell lines were grown in full medium (FM): DMEM supplemented with 20% fetal calf serum and 4.8 mM L-glutamine. To induced autophagy, cells were washed in and incubated in Earle's balanced salt solution (EBSS) for two hours. Where indicated, cells were treated with: 100 nM bafilomycin A1 (Calbiochem), or 100 nM Wortmannin (Calbiochem). FIP200$^{-/-}$ and matched wild type immortalized MEF cell lines were a kind gift from Jun-Lin Guan (Gan et al., 2006), and Atg16$^{-/-}$ and matched immortalized MEF cell lines were kind gift from Shizuo Akira (Saitoh et al., 2008), Atg5$^{-/-}$ and matched wild type immortalized MEF cell lines were a kind gift from Noboru Mizushima (Kuma et al., 2004), Atg3$^{-/-}$ and matched wild type immortalized MEF cell lines were a kind gift from Masaaki Komatsu(Sou et al., 2008). The 293/GFP-LC3 cells are as previously described (Chan et al., 2007). HEK293 cell lines stably expressing GFP, GFP-WIPI1a and GFP-WIPI2b were established by transient transfection of the pEGFP-constructs, selection with G418 (800 μg/mL) and cloned by limiting dilution and then selected for low level expression by FACs sorting before being maintained in the presence of G418 at 400μg/mL. GFP-LC3 MEFs were kindly provided by Fiona McAlpine (LRI, CRUK). HEK239A/DFCP1 (201 cells) cells were provided by N. T. Ktistakis (Babraham Institute, Cambridge, UK).

Lipofectamine 2000 (Invitrogen) was used according to the manufacturer's instructions for transient transfection of HeLa, HEK293A and derivative cells. DNA plasmids were used at a concentration of 1μg/mL of transfection mix. For RNA interference of HeLa, HEK293A and derivate cells, cells were transfected with the relevant siRNA oligo using Oligofectamine (Invitrogen), followed by a second transfection using Lipofectamine 2000 (Invitrogen) the following day, both used according to the manufacturer's instructions. Final concentration of siRNA oligos was 50 nM. siRNA Oligos used: Darmacon: D-001220-01 (RISC-Free, control), L-021033-01 (Atg16L1), LU-021117-00 (RB1CC1/FIP200), Qiagen: Hs_WIPI2_4 (WIPI2). Knock-down and rescue experiments were performed by using oligofectamine with siRNA on day one, followed by lipofectamine with siRNA and DNA on day two. Rescue assay was performed 48 hours after lipofectamine treatment. For transfecton of MEF cells, jetPRIME (Polyplus-

transfection) was used according to the manufacture's instructions. DNA plasmids were used at a concentration of 1μg/mL of transfection mix. MEF rescue assays were performed 24 hours after transient transfection with jetPRIME.

pEGFP-C1-WIPI1a construct (Accession No. AY691424) was a gift from Tassula Proikas-Cezanne (University of Tuebingen, Germany) and mutated to agree with gene bank sequence BC039867 (Val to Ala at amino acid position 81). GFP-WIPI4 was a gift from Noboru Mizushima (University of Tokyo). GFP-LC3, FLAG-Atg16L1 WT, 1-265 and 79-623 constructs were a gift from Tamotsu Yoshimori (Osaka Univeristy). FLAG-Atg16L2 construct was a gift from Mitsunori Fukuda (Tohoku University). pEGFP-WIPI2a and WIPI2b were generated by PCR amplification, respectively. The lipid-binding mutant (FRRG to FTTG) and point mutants of GFP-WIPI2b, and point mutants and truncations of FLAG-Atg16L1 were generated using QuikChange Site-directed Mutagenesis Kit (Stratagene). WIPI2-CAAX constructs were generated using PCR amplification and site-directed mutagenesis. Primers, see Table S1.

Mouse monoclonal antibodies: anti-LC3 (Nanotools, 0231-100/LC-3-5F10), anti-myc 9E10 (CRUK), anti-Atg16L1 (MBL, M150-3B), anti-FLAG-M2 (Sigma, F3165), anti-GFP (CRUK, 3E1), anti-HA (Covance, MMS-101R), anti-LAMP1 (BD Biosciences, 555801) anti-p62 (BD Biosciences, 610833), anti-ubiquitin (MBL, D058-3), and anti-WIPI2 (polson et al.). Rabbit polyclonal anti-Atg16L (Cosmo Bio, TMD-PH-AT16), anti-FLAG (Sigma, F7425), anti-LC3 (Abcam, ab48394), anti-β actin (Abcam, ab8227), anti-Atg5 (Cosmobio, TMD-PH-At5), anti-Atg12 (Cell signalling, #2010), anti-FIP200 (Bethyl Laboratories, A301-536A), anti-RFP (MBL, PM005), anti-WIPI1(Polson et al., 2010) and anti-WIPI2(Polson et al., 2010). Guinea pig polyclonal anti-p62 (Progen, cat. No. GP62-C). Chicken polyclonal anti-calreticulin (ab2908). Antibodies were used at manufacturers suggested concentrations. Secondary antibodies used for IF were anti-rabbit IgG Alexa Fluor 488, and 555, anti-mouse IgG Alexa Flour 488, 555, and 647, and anti-guinea pig IgG Alexa Fluor 555. HRP-conjugated secondary antibodies used for WB were from GE Healthcare.

Protein complex purification and Mass Spectroscopy

Fed or starved cell pellets washed in PBS were lysed in 10 mM Tris-HCl pH7.5, 150 mM NaCl, 0.5 mM EDTA, 0.5 % NP40, 2x Complete protease inhibitor (Roche,), 1x PhosSTOP (Roche) and the lysate clarified by high speed centrifugation (20,000xg 10 min). Lysates

were diluted to 0.2% NP-40 and incubated with GFP-Trap® beads at 4°C for 1 hour. Pelleted beads were washed 3 times with 10 mM Tris-HCl pH7.5, 150 mM NaCl, 0.5 mM EDTA, 1 % NP40, 1x Complete protease inhibitor, 1x PhosSTOP and protein complexes eluted with 2x Laemmli sample buffer at 65°C for 10 min.

Immunoprecipitation:

Endogenous immunoprecipiation: cells were permeabilised using TNTE buffer (1% triton TX-100, 20 mM Tris HCL, pH7.5, 150 mM NaCl, 5 mM EDTA, 1 X Complete protease inhibitor) and the lysates used for immunoprecipitation with monoclonal anti-WIPI2 (A2A) overnight before washing with TNTE. Crosslinker was cleaved by boiling the sample in Laemmli sample buffer containing 50 mM DTT and boiled for 10 minutes before resolving by SDS-PAGE (4-12% BisTris NUPAGE gels, Invitrogen). GFP-tagged proteins were immunoprecipitated using GFP-Trap® beads as for mass spectrometry, but with 500 mM NaCl in the wash buffer and no PhosSTOP in any buffer. FLAG-tagged proteins were immunoprecipitated using anti-FLAG M2 affinity gel (Sigma) for one hour with the same lysis and wash buffers as used for GFP-Trap®.

In vitro translation

For binding assays using *in vitro* translated proteins, cells overexpressing GFP-tagged proteins were used for GFP- Trap®as described as above. The GFP-Trap® product was then mixed with in vitro translated FLAG-tagged protein. Mixtures were incubated for one hour before washing with GFP-Trap® wash buffer. The product was run on SDS-PAGE (4-12% BisTris NUPAGE gels, Invitrogen) before Coomasssie Blue staining, drying and auto-radiation. *In vitro* translated protein was produced using TNT® Quick Coupled Transcription/Translation System (Promega) and EasyTag™ L-[^{35}S]-methonine (PerkinElmer).

Confocal microscopy

Cells were grown on coverslips, fixed with 3% paraformaldehyde for 20 minutes before permeablisation with either 50 ug/mL digitonin (endogenous Atg16 staining), or with room temperature methanol (endogenous LC3 staining) for five minutes. Coverslips were blocked in 5% BSA (Roche) for one hour, incubated with primary antibody in 1% BSA for one hour, washed in 1% BSA, incubated with secondary antibody in 1% BSA for

one hour, before final washing with PBS and water. LC3 puncta formation was assessed by Imaris image analysis software. Salmonella experiments were quantified using Imaris-assisted manual counting.

Correlative Light and Electron Microscopy (CLEM)

CLEM was performed as previously described (Razi and Tooze, 2009). In Fig. 1d-e, Atg16L1 MEFs were fixed with PFA 2% + Glut 2% in PBS, followed by Digitonin permilization for 5 mins (50ng/ul), then washed twice in 10% BSA for 2x5min. Cells were stained with anti-WIPI2 antibody, and secondary as described above. HEK293 cells expressing GFP-WIPI2b RERE (Fig. 4i, j), or MCF7 cells expressing mCherry-WIPI2b-CAAX and GFP-LC3 (Fig. 6e) were processed and imaged as previously described (Orsi et al., 2012).

Live cell imaging

Live cell imaging of HEK293 cells stably expressing GFP-WIPI2b co-transfected with mCherry-Atg16L1. Cells were placed into EBSS at 37°C and immediately imaged using an UltraVIEW Perkin-Elmer spinning disk confocal microscope. Images were processed using ImageJ and MetaMorph software.

Modeling

A model for human WIPI2b (14-377) was made using the X-ray structure of the *Kluyvelomyces marxianus* Hsv2 homologue ((Watanabe et al., 2012); 3vu4.pdb) with the iterative threading assembly refinement (I-TASSER) (Roy et al., 2010). The model geometry was refined using COOT (Emsley et al., 2010). Final backbone geometries were 100% in allowed and preferred regions of the Ramachandran plot, all sidechain geometries were acceptable and no clashes were detected. Structural alignment of the WIPI2b model with 3vu4:A using TMalign (Zhang and Skolnick, 2005) revealed that the model aligned to 316 of 319 residues in 3vu4:A, with an RMSD <0.89. An ab initio model for human Atg16L1 (207-246) was using I-TASSER and model geometry refined using COOT.

Statistical analysis

Performed using Prism 6 software as detailed in the figure legends.

Primers used in this study

Primer	Sequence
WIPI2b siRES-SDM-1	GTGGGTGCCGCCGACGGGTATCTTTACATGTACAAC
WIPI2b siRES-SDM-2	GTGGGTGCCGCCGATGGTTATCTTTACATGTACAAC
HA-WIPI2CAAX-forward	ATGAATTCACCATGTACCCATACGATGTTCCAGATTACGCTAACCTGGCGAGCCAGAGCGGG
Reverse-WIPI2CAAX	AACTTAAGCTTTCACATAATTACAC
mCherry-WIPI2CAAX-forward	ATCTCGAGGGAACCTGGCGAGCCAGAGCGGG
Atg16L1 mus 1-230 SDM	GAAGCAGCAAAGGAATAGCCTCTACCTGTTGAAC
Atg16L1 mus 1-242 SDM	GATGACATTGAAGTCTAGATTGTGGATGAGACC
Atg16L1 mus 1-207 SDM	CAATCGCCTCAATGCATAGGAGAATGAGAAGGAC
WIPI2b-R108E	GCTGTGAAGCTCAACgagCAGAGGCTGATAGTATG
WIPI2b-R125E	CATCCACAACATTgagGACATGAAGGTGCTGC
Atg16L1 mus E208R	CCAATCGCCTCAATGCAAGGAATGAGAAGGACTCC
Atg16L1 mus D212R	GCAGAGAATGAGAAGcgcTCCAGGAGGCGTCAAGC

Atg16L1 mus E226R	CTGCAGAAGGAGCTTGCAagaGCAGCAAAGGAACCTCTAC
Atg16L1 mus E230R	GAGCTTGCAGAAGCAGCAAAGagaCCTCTACCTGTTGAAC
Atg16L1 mus E235R	CAAAGGAACCTCTACCTGTTagaCAGGATGATGACATTGAAG
Atg16L1 mus D237R	CCTCTACCTGTTGAACAGcgtGATGACATTGAAGTCATTG
Atg16L1 mus D238R	CTACCTGTTGAACAGGATcgtGACATTGAAGTCATTGTGG
Atg16L1 mus D239R	CCTGTTGAACAGGATGATcgcATTGAAGTCATTGTGGATG
Atg16L1 mus E241R	GAACAGGATGATGACATTcgtGTCATTGTGGATGAGACC
dCAAX SDM	CCATGATTCTTCGGACTGACtgaAGAAAAGATGGTAAAAAGAAG
Atg16L1 mus E226R E230R	GAGCTTGCAagaGCAGCAAAGagaCCTCTACCTGTTGAAC
Atg16L1 mus D237R D239R	CCTGTTGAACAGcgtGATcgcATTGAAGTCATTGTGGATG
Atg16L1 mus E246R	CATTGAAGTCATTGTGGATaggACCTCAGACCACACAGAAG
Atg16L1 mus D249R	CATTGTGGATGAGACCTCAcgcCACACAGAAGAGACCTCTC
Atg16L1 mus E253R	GACCTCAGACCACACAGAAcggACCTCTCCCGTCCGAGCTG
WIPI2 mut FTTG	GGACAAAAACTCTTTGAGTTTACGACAGGAGTAAAGAGGTGCGTG

WIPI1 dCT	ACAGCTTGCTTGGCTCAtaaACAACAGAAGAGAATAAAG
WIPI2 dCT	CACCGGCTGGACGGCAGTtaaGAAACGACCAATGAGATC

References

CONSORTIUM, U. 2013. Update on activities at the Universal Protein Resource (UniProt) in 2013. *Nucleic Acids Res.*

EMSLEY, P., LOHKAMP, B., SCOTT, W. G. & COWTAN, K. 2010. Features and development of Coot. *Acta Crystallogr D Biol Crystallogr.* International Union of Crystallography.

GAN, B., PENG, X., NAGY, T., ALCARAZ, A., GU, H. & GUAN, J.-L. 2006. Role of FIP200 in cardiac and liver development and its regulation of TNFα and TSC–mTOR signaling pathways. *The Journal of Cell Biology,* 175**,** 121-133.

KUMA, A., HATANO, M., MATSUI, M., YAMAMOTO, A., NAKAYA, H., YOSHIMORI, T., OHSUMI, Y., TOKUHISA, T. & MIZUSHIMA, N. 2004. The role of autophagy during the early neonatal starvation period. *Nature,* 432**,** 1032-6.

LYSKOV, S. & GRAY, J. J. 2008. The RosettaDock server for local protein-protein docking. *Nucleic Acids Res.*

MCALPINE, F., WILLIAMSON, L., TOOZE, S. A. & CHAN, E. Y. W. 2013. Regulation of nutrient-sensitive autophagy by uncoordinated-51 like kinases 1 and 2. *Autophagy,* 9**,** 361-373.

ORSI, A., RAZI, M., DOOLEY, H., ROBINSON, D., WESTON, A., COLLINSON, L. & TOOZE, S. 2012. Dynamic and transient interactions of Atg9 with autophagosomes, but not membrane integration, is required for autophagy. *Mol Biol Cell,* 23**,** 1860-1873.

PIERCE, B. G., HOURAI, Y. & WENG, Z. 2011. Accelerating protein docking in ZDOCK using an advanced 3D convolution library. *PLoS ONE.*

RAZI, M. & TOOZE, S. A. 2009. Correlative light and electron microscopy. *Methods Enzymol,* 452**,** 261-75.

ROY, A., KUCUKURAL, A. & ZHANG, Y. 2010. I-TASSER: a unified platform for automated protein structure and function prediction. *Nature protocols.*

SOU, Y.-S., WAGURI, S., IWATA, J.-I., UENO, T., FUJIMURA, T., HARA, T., SAWADA, N., YAMADA, A., MIZUSHIMA, N., UCHIYAMA, et al. 2008. The Atg8 Conjugation System Is Indispensable for Proper Development of Autophagic Isolation Membranes in Mice. *Mol. Biol. Cell,* 19**,** 4762-4775.

WATERHOUSE, A. M., PROCTER, J. B., MARTIN, D. M., CLAMP, M. & BARTON, G. J. 2009. Jalview Version 2--a multiple sequence alignment editor and analysis workbench. *Bioinformatics,* 25**,** 1189-91.

ZHANG, Y. & SKOLNICK, J. 2005. TM-align: a protein structure alignment algorithm based on the TM-score. *Nucleic Acids Res.*
