# Supplementary figures and images for: WIPI2 Links LC3 Conjugation with PI3P, Autophagosome Formation, and Pathogen Clearance by Recruiting Atg12–5-16L1
Source: Mol Cell. 2014 Jul 17;55(2):238–52. doi: 10.1016/j.molcel.2014.05.021 (PMC4104028; doi:10.1016/j.molcel.2014.05.021)

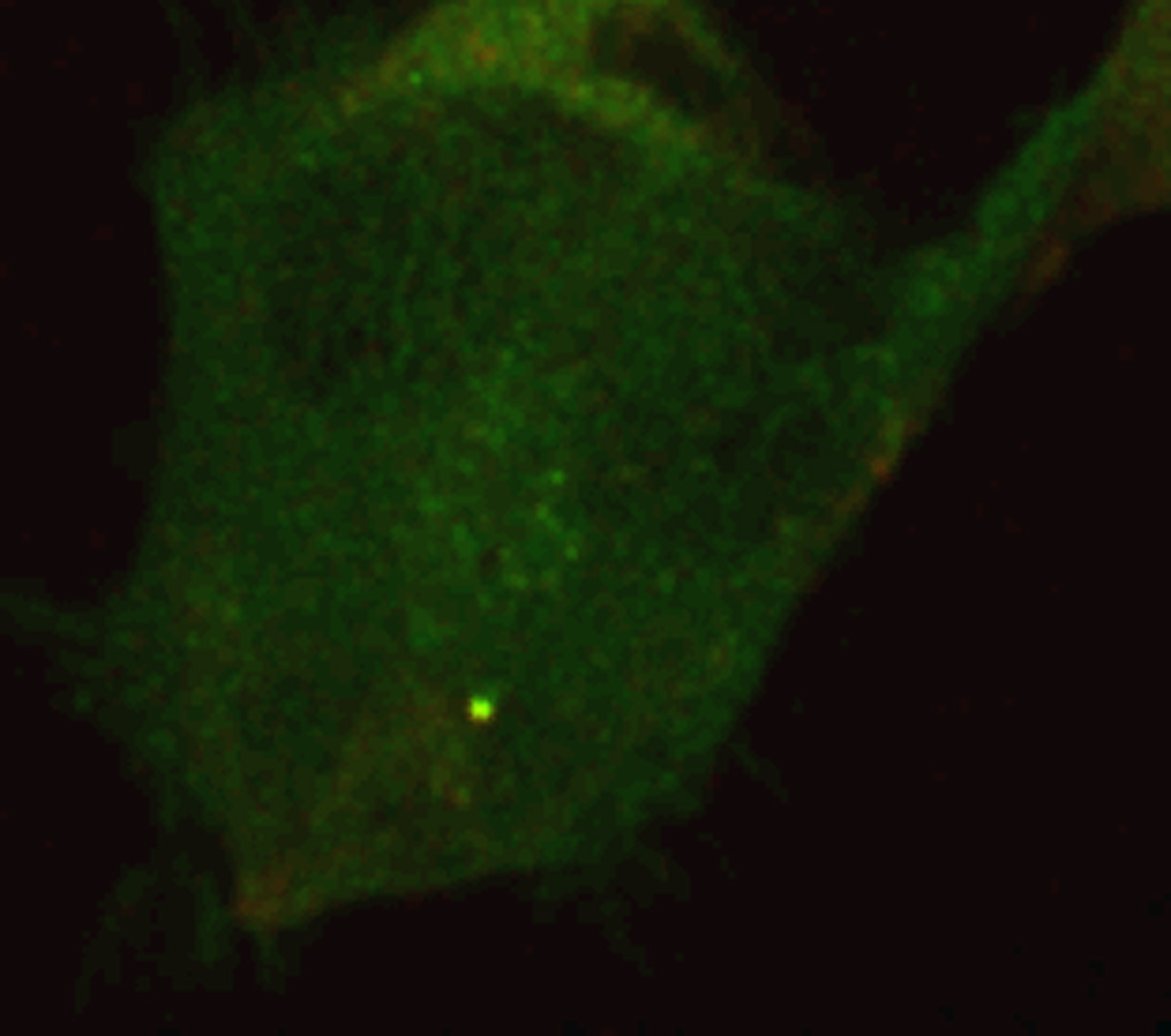

Supplement: Movie S1. mCherry-Atg16L1 Is Recruited to GFP-WIPI2b Puncta during Amino Acid Starvation, Related to Figure 1 — Live-cell imaging of HEK293A cells stably expressing GFP-WIPI2b cotransfected with mCherry-Atg16L1. Cells were placed into EBSS at 37°C and immediately imaged using an UltraVIEW PerkinElmer Spinning Disk Confocal Microscope. Arrows mark the WIPI2b-positive structures shown in Figure 1K. The arrows appear in the sequence shown in Figure 1K (top, middle, and bottom panels). [file mmc2.jpg]
