# Supplementary material for: WIPI2 Links LC3 Conjugation with PI3P, Autophagosome Formation, and Pathogen Clearance by Recruiting Atg12–5-16L1
Source: Mol Cell. 2014 Jul 17;55(2):238–52. doi: 10.1016/j.molcel.2014.05.021 (PMC4104028; doi:10.1016/j.molcel.2014.05.021)
Supplement: Document S2. Article plus Supplemental Information [file mmc3.pdf]

Molecular Cell
Article

WIPI2 Links LC3 Conjugation with PI3P, Autophagosome Formation, and Pathogen Clearance by Recruiting Atg12–5-16L1

Hannah C. Dooley,[1] Minoo Razi,[1] Hannah E.J. Polson,[1] Stephen E. Girardin,[2] Michael I. Wilson,[3] and Sharon A. Tooze[1,*]
[1]London Research Institute, Cancer Research UK, 44 Lincolns Inn Fields, London WC2A 3LY, UK
[2]Department of Laboratory Medicine and Pathobiology, University of Toronto, Toronto, ON M5S 1A8, Canada
[3]The Babraham Institute, Babraham Research Campus, Cambridge CB22 3AT, UK
*Correspondence: sharon.tooze@cancer.org.uk
http://dx.doi.org/10.1016/j.molcel.2014.05.021
This is an open access article under the CC BY license (http://creativecommons.org/licenses/by/3.0/).

SUMMARY

Mammalian cell homeostasis during starvation depends on initiation of autophagy by endoplasmic reticulum-localized phosphatidylinositol 3-phosphate (PtdIns(3)P) synthesis. Formation of double-membrane autophagosomes that engulf cytosolic components requires the LC3-conjugating Atg12–5-16L1 complex. The molecular mechanisms of Atg12–5-16L1 recruitment and significance of PtdIns(3)P synthesis at autophagosome formation sites are unknown. By identifying interacting partners of WIPIs, WD-repeat PtdIns(3)P effector proteins, we found that Atg16L1 directly binds WIPI2b. Mutation experiments and ectopic localization of WIPI2b to plasma membrane show that WIPI2b is a PtdIns(3)P effector upstream of Atg16L1 and is required for LC3 conjugation and starvation-induced autophagy through recruitment of the Atg12–5-16L1 complex. Atg16L1 mutants, which do not bind WIPI2b but bind FIP200, cannot rescue starvation-induced autophagy in Atg16L1-deficient MEFs. WIPI2b is also required for autophagic clearance of pathogenic bacteria. WIPI2b binds the membrane surrounding *Salmonella* and recruits the Atg12–5-16L1 complex, initiating LC3 conjugation, autophagosomal membrane formation, and engulfment of *Salmonella*.

INTRODUCTION

Autophagy is a conserved degradation pathway, present in all eukaryotes, required for cell survival during starvation and cell homeostasis. In higher eukaryotes, autophagy is also required for development, immunity, and combating infection (Choi et al., 2013). During macroautophagy, here called autophagy, double-membrane phagophores (isolation membranes) expand and sequester cytosolic components, forming autophagosomes that fuse with endosomes and lysosomes. Phagophores arise from endoplasmic reticulum (ER)-derived omegasomes (Axe et al., 2008), and during autophagosome expansion, membrane from other subcellular compartments (Golgi, mitochondria, and plasma membrane) is probably incorporated (Lamb et al., 2013).

The formation of autophagosomes requires autophagy-related (Atg) proteins, first identified in yeast (Mizushima et al., 2011), which are recruited in a sequential manner (Itakura and Mizushima, 2010). The first Atg protein complex to respond to upstream signals, in particular inactivation of mammalian target of rapamycin complex 1 (mTORC1), is the Unc-51-like kinase (ULK) complex. Activation of ULK1/ULK2 and subsequent phosphorylation of ULK complex members Atg13 and FIP200 initiates autophagy. The class III Vps34 lipid kinase, part of the Beclin1 complex, is then activated (Wirth et al., 2013), translocates to ER sites, and produces a pool of phosphatidylinositol 3-phosphate (PtdIns(3)P). Live-cell imaging has shown that the ULK complex translocates first to the ER formation site, but PtdIns(3)P produced by the Beclin1 complex is required for stabilization of the ULK complex at the omegasome (Karanasios et al., 2013, Koyama-Honda et al., 2013).

PtdIns(3)P at the omegasome facilitates recruitment of PtdIns(3)P effectors such as DCFP1 and the WIPI (WD-repeat PtdIns(3)P effector protein) family of proteins (Karanasios et al., 2013, Koyama-Honda et al., 2013, Polson et al., 2010), the function of which is unknown. The final Atg proteins recruited prior to LC3 lipidation are the Atg12–5-16L1 complex and the Atg3-LC3 conjugate. In the Atg12–5-16L1 complex, Atg16L1 binds to Atg5, which is conjugated to Atg12. This complex acts as an E3-like enzyme to recruit the E2-like protein Atg3, conjugated to LC3-I, to the omegasome. Atg3 binds to and is activated by Atg12 (Sakoh-Nakatogawa et al., 2013), facilitating conjugation of the LC3 to phosphatidylethanolamine (PE), thus converting LC3-I to LC3-II. Therefore, the site of Atg12–5-16L1 complex recruitment determines the site of LC3-II formation (Fujita et al., 2008). The LC3 family (Atg8 in yeast) is required for phagophore expansion, closure, and cargo recruitment (Slobodkin and Elazar, 2013). The mechanism by which the Atg12–5-16L1 complex is recruited to membranes is unknown. While Atg16L1 binds FIP200 (Gammoh et al., 2013, Nishimura et al., 2013, Fujita et al., 2013), recruitment of Atg12–5-16L1 depends on PtdIns(3)P formation (Itakura and Mizushima, 2010). However, no PtdIns(3)P binding protein has been implicated in recruiting the Atg12–5-16L1 complex, and the molecular mechanism

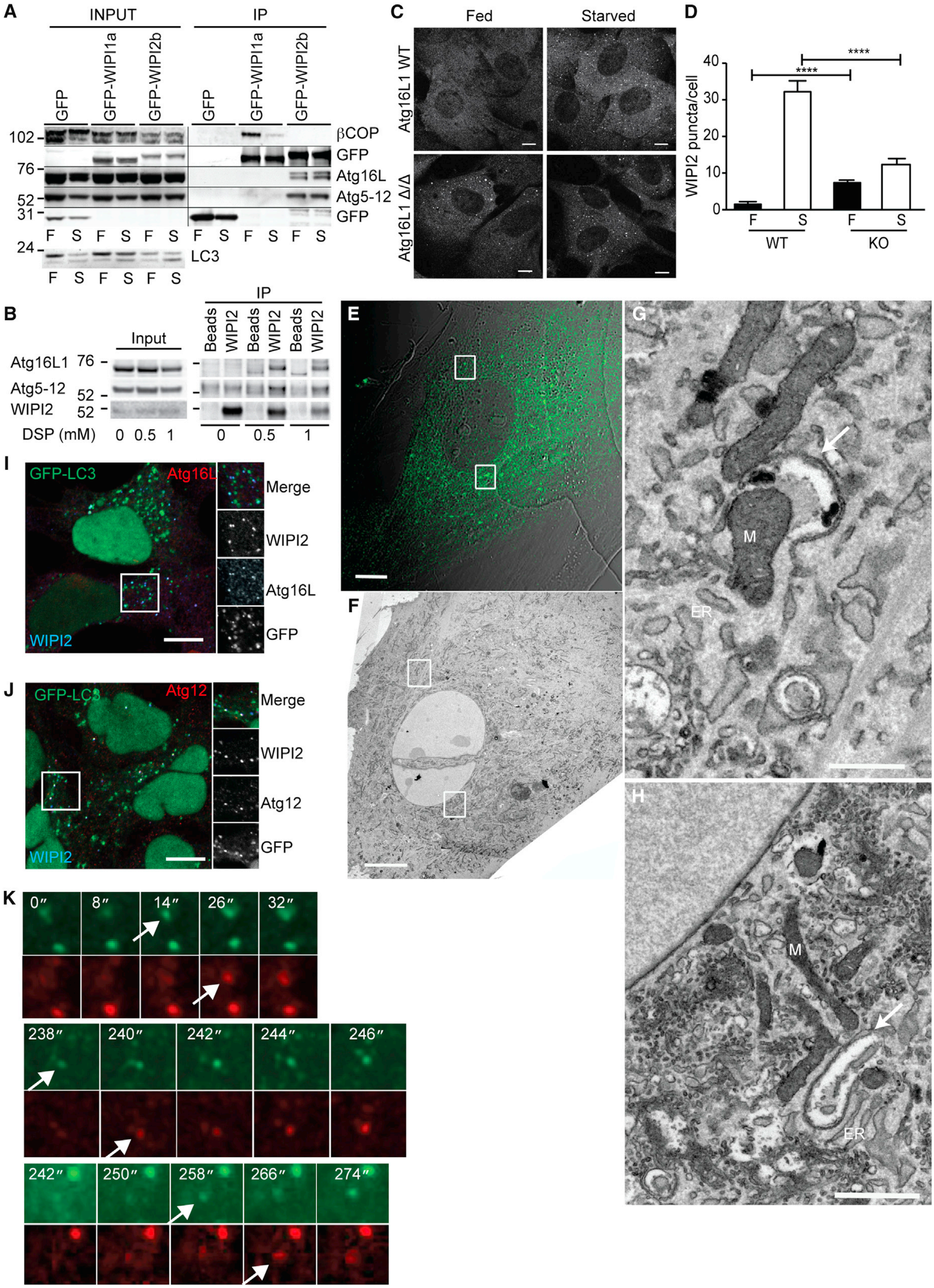

(legend on next page)

underlying the requirement for PtdIns(3)P production during phagophore formation in mammalian autophagy remains unclear.

We have addressed the function of the PtdIns(3)P effector WIPI proteins by identifying WIPI1 and WIPI2 interacting proteins. Our comparative analysis revealed that Atg16L1 directly interacts with WIPI2b, and we identified the residues required for interaction between WIPI2b and Atg16L1. We demonstrate that WIPI2b acts immediately upstream of Atg16L1 and is responsible for Atg12–5-16L1 recruitment to PtdIns(3)P-positive omegasomes, resulting in LC3 lipidation and starvation-induced autophagy. We also show that Atg16L1 mutants able to bind FIP200, but not WIPI2b, cannot rescue starvation-induced autophagy in Atg16L1-deficient mouse embryonic fibroblasts (MEFs). Finally, we show that WIPI2b function is also required for the innate immune response to *Salmonella* Typhimurium infection. WIPI2b binds the phagophore membrane surrounding *Salmonella*, recruits Atg16L1, initiating LC3 lipid conjugation, autophagosomal membrane formation, and engulfment of *Salmonella*, and restricts bacterial proliferation.

RESULTS

WIPI2b Binds Atg16L1

We have shown that WIPI2 is required for starvation-induced autophagy (Polson et al., 2010). WIPI1, a closely related family member, also regulates autophagy (Proikas-Cezanne et al., 2004, Polson et al., 2010). In cells expressing WIPI1 and WIPI2, loss of WIPI1 increases autophagy, suggesting that WIPI1 may have an inhibitory role (Polson et al., 2010). WIPI2 has five isoforms (a, b, c, d, and e) that are differentially recruited to autophagosomes upon amino acid starvation (Mauthe et al., 2011). WIPI2a and WIPI2c are not recruited to the membrane upon starvation, and WIPI2a does not colocalize with DFCP1 (see below). To identify proteins that interact with WIPI2 and/or WIPI1, we used human embryonic kidney 293 (HEK293) cell lines stably expressing GFP, GFP-WIPI1a, and GFP-WIPI2b, which responded to amino acid starvation and formed wortmannin-sensitive (PtdIns(3)P-dependent) WIPI puncta, for a mass spectroscopy screen for interactors (Figures S1A–S1D, available online).

A distinct set of proteins was identified in the GFP-WIPI1a and GFP-WIPI2b pull-downs (Figure S1E). The most abundant interacting proteins for GFP-WIPI1a were coatomer subunits, validated by immunoblot (Figure 1A). GFP-WIPI2b bound Atg5 and Atg16L1, and the interaction between WIPI2b, but not WIPI1a, and the Atg16L1 complex was validated by immunoblot (Figure 1A). The interaction of endogenous WIPI2 with Atg16L1 (Figure 1B) was confirmed using a crosslinker to stabilize the interaction. This low-affinity or transient interaction between WIPI2 and Atg16L1 is reminiscent of that between Atg16L1 and FIP200, detected after crosslinking (Nishimura et al., 2013) or in Atg3$^{-/-}$ MEFS (Gammoh et al., 2013).

We chose to focus on the interaction of the Atg16L1 complex with WIPI2b, as the PtdIns(3)P-dependent recruitment of Atg12–5-16L1 to the omegasome and phagophore could be the function of WIPI2 in autophagy. In support of our hypothesis that WIPI2 acts upstream of Atg16L1, WIPI2 puncta are seen in Atg16L1$^{\Delta/\Delta}$ MEFs in both fed and starved conditions (Figures 1C and 1D). These puncta are phagophores and open autophagosomes (Figures 1E–1H).

Endogenous WIPI2 colocalized with Atg16L1 (Figure 1I) and Atg12 (Figure 1J) on both GFP-LC3-negative and -positive autophagosomes. Note that myc-WIPI2b colocalization with DFCP1 on omegasomes is starvation and PtdIns(3)P-binding dependent, while WIPI2a is not detected on omegasomes (Figure S1E) (Mauthe et al., 2011). To probe the relationship between WIPI1a and WIPI2b, we tested if GFP-WIPI1a and GFP-WIPI2b were present on Atg16L1-positive autophagosomes. In cells expressing low levels of GFP-WIPI1a, where no displacement of endogenous WIPI2 occurred (Polson et al., 2010), the GFP-WIPI1a-positive puncta did not contain Atg16L1 in contrast to GFP-WIPI2b (Figures S1E and S1H). GFP-stable cell lines have GFP-negative, WIPI2-Atg16-positive autophagosomes (Figure S1I). Under starvation, live-cell imaging of GFP-WIPI2b and mCherry-Atg16L1 revealed that Atg16L1 translocated to GFP-WIPI2b-positive puncta (Figure 1K and Movie S1), supporting our proposed sequential recruitment of WIPI2b to an omegasome-derived pool of PtdIns(3)P followed by the Atg16L1 complex.

A Region in Atg16L1 Not Conserved in Atg16L2 Binds WIPI2b

Atg16L1 is the protein through which the Atg12–5-16L1 complex is recruited to the forming autophagosome (Fujita et al., 2008), and we propose that by binding Atg16L1, WIPI2b is recruiting the Atg12–5-16L1 complex. To map the binding site, we used deletion constructs of Atg16L1 (Figure 2A). Previous work has

Figure 1. WIPI2 Binds and Colocalizes with the Atg12–5-16L1 Complex

(A) HEK293A cell lines stably expressing GFP, GFP-WIPI1a, and GFP-WIPI2b were incubated in full medium (F) or starvation medium (EBSS) (S) for 2 hr before being used for GFP-TRAP pull-down.

(B) WIPI2 was immunoprecipitated from HEK293A cells treated with DSP, at the indicated concentrations, before immunoblotting.

(C) WT or Atg16L1$^{\Delta/\Delta}$ MEFs in fed medium or starved for 2 hr in EBSS were fixed and labeled with an anti-WIPI2 antibody. Scale bars, 10 μm.

(D) WIPI2 puncta in (C) were counted, and a statistical analysis of WIPI2 puncta was performed using an unpaired Student's t test. *$p < 0.05$. SEM for n = 3.

(E–H) CLEM (correlative light and electron microscopy) of endogenous WIPI2 in Atg16L1$^{\Delta/\Delta}$ MEFs.

(E) Merged phase and confocal section of Atg16L1$^{\Delta/\Delta}$ cells labeled with anti-WIPI2 antibody. Scale bar, 10 μm.

(F) Low-magnification TEM of cell in (E). Scale bar 10μm.

(G and H) High magnification of boxed regions in (E) and (F). Top box and bottom box indicate panels (G) and (H), respectively. Arrows indicate open phagophores. M, mitochondria; ER, endoplasmic reticulum. Scale bars, 1 μm (G) and 0.5 μm (H).

(I and J) 2GL9 cells (GFP-LC3 HEK293 cells; Chan et al., 2007) were starved for 2 hr before visualization using indicated antibodies. Scale bars, 10 μm.

(K) Live-cell imaging demonstrates that Atg16L1 translocates to WIPI2b-positive puncta in starvation. HEK293 cells expressing GFP-WIPI2b and mCherry-Atg16L1 were starved in EBSS and imaged every 2 s using a spinning disk microscope. See also Figure S1 and Movie S1.

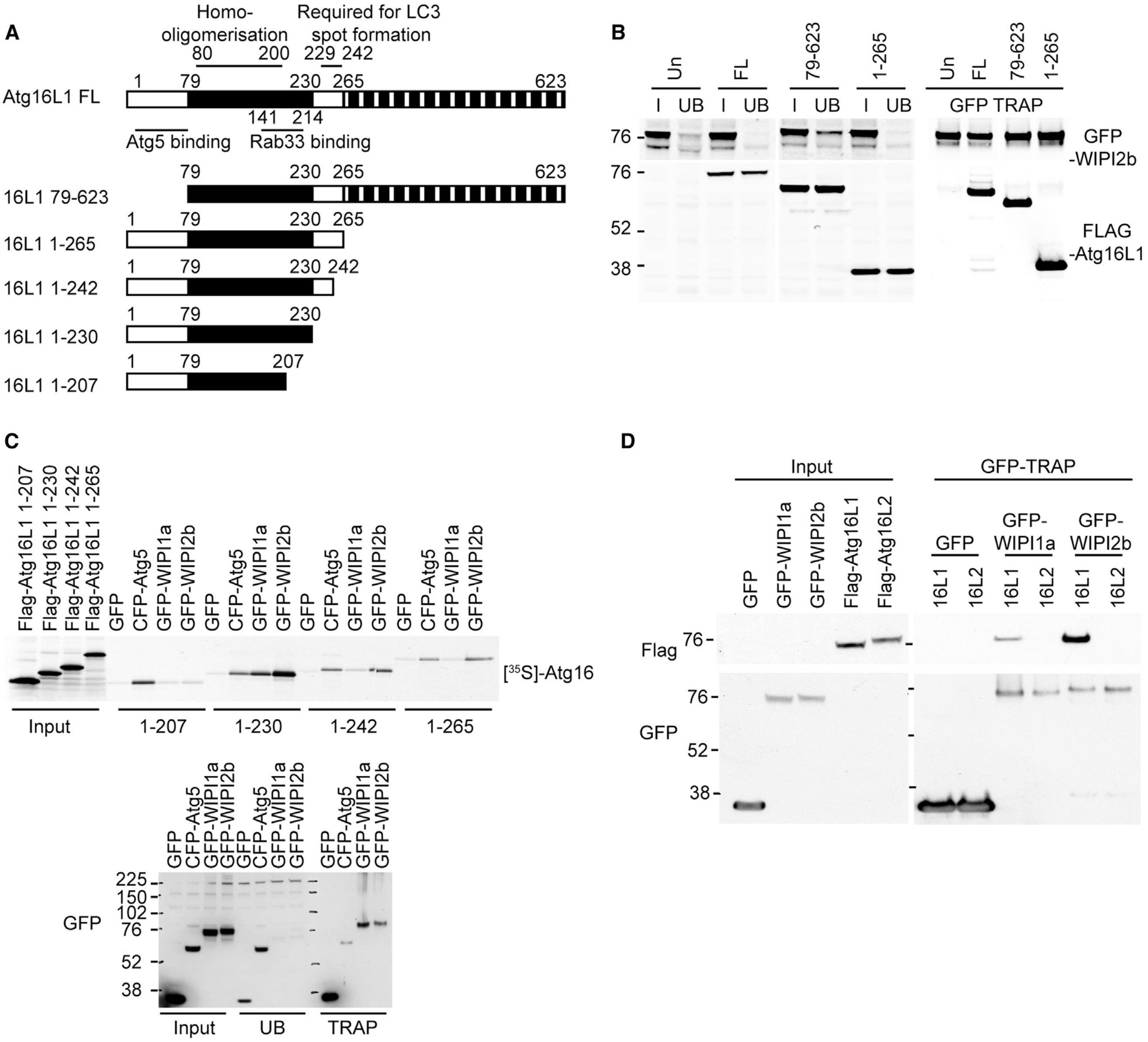

Figure 2. Interaction of Atg16L1 with WIPI2 Requires 207–242, a Domain Not Functionally Conserved in Atg16L2

(A) Scheme of mouse Atg16L1 and deletion mutants showing the N-terminal Atg5 interacting domain (white box), a coiled-coil domain (black box), and a WD propeller-repeat domain (striped box).

(B) Untransfected (UN) or FLAG-Atg16L1 full-length (FL), 79–623, or 1–265 constructs were expressed in HEK293A cells stably expressing GFP-WIPI2b and immunoprecipitated using GFP-TRAP. Tags were visualized by immunoblotting. I, input; UB, unbound.

(C) GFP, CFP-Atg5, GFP-WIPI1a, and GFP-WIPI2b were transiently expressed in HEK293A cells. GFP-tagged proteins were isolated using GFP-TRAP and incubated with in vitro translated ^{35}S-labeled FLAG-Atg16L1 constructs 1–265, 1–242, 1–230, and 1–207 before washing and analysis by autoradiography. Protein expression was validated by immunoblot (bottom panel).

(D) Lysates from HEK293A cells transiently expressing GFP, GFP-WIPI1a, or GFP-WIPI2b were mixed with lysates from HEK293A cells transiently expressing FLAG-Atg16L1 or Atg16L2. Protein complexes were immunoprecipitated using GFP-TRAP, followed by immunoblot. See also Figure S2 and Table S1.

identified the domains of Atg16L1, which bind Atg5 (Mizushima et al., 2003) and Rab33b (Itoh et al., 2008) and mediate dimerization (Itoh et al., 2008) and LC3-spot formation (Ishibashi et al., 2011). Deletion of the N-terminal Atg5 binding domain (1–78 aa) or C-terminal WD domain (266–623 aa) had no effect on the Atg16L1-WIPI2b interaction in GFP pull-down experiments (Figure 2B). We concluded that WIPI2b was interacting with Atg16L1 between 79 and 265. A similar result was obtained using human Atg16L1 constructs (Figures S2A and S2B). We refined the interaction site using truncation mutants of FLAG-Atg16L1 constructs, with GFP and CFP-Atg5 as controls, and GFP-WIPI1a or GFP-WIPI2b (Figures 2C and S2C). GFP-WIPI2b

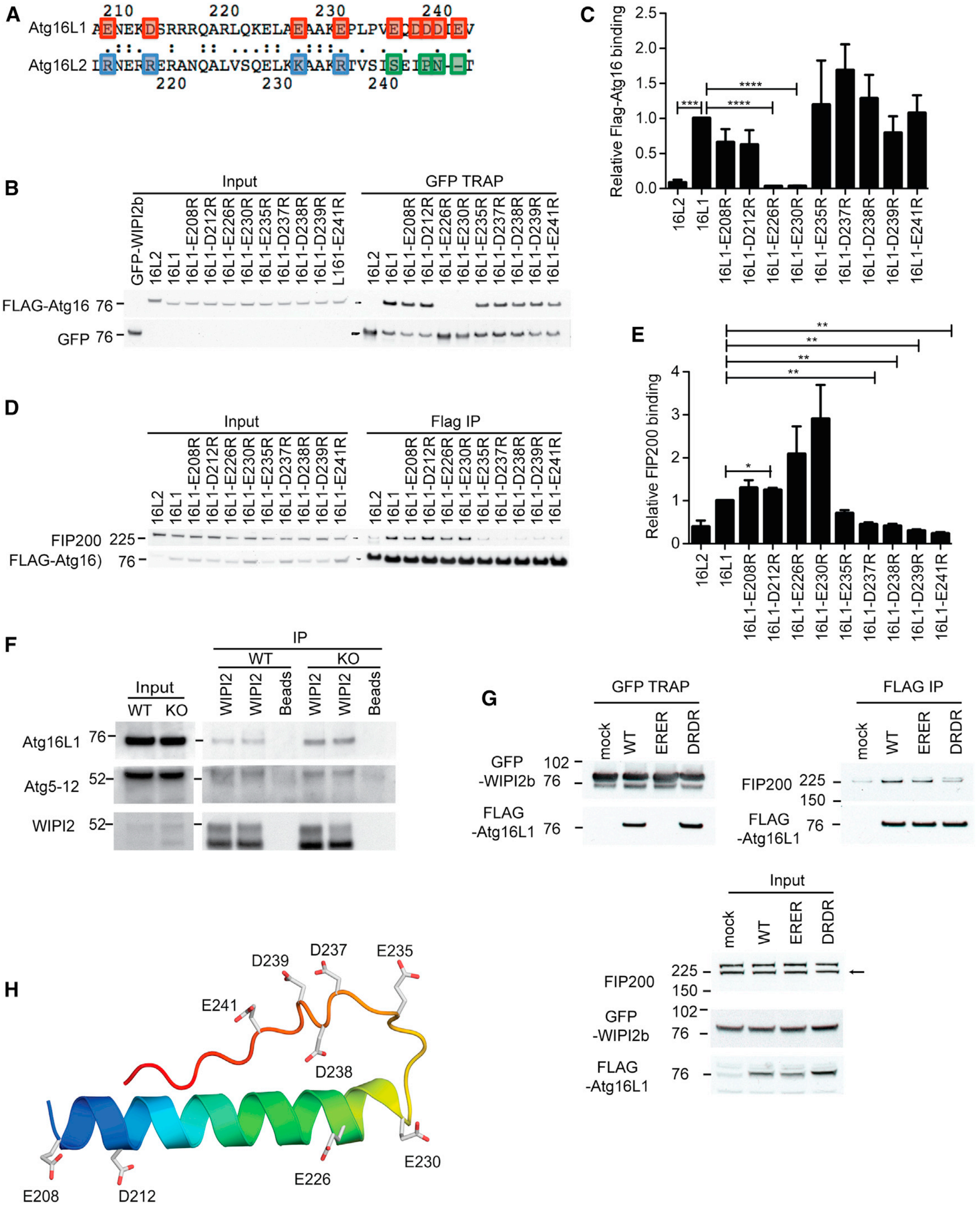

Figure 3. WIPI2b and FIP200 Bind in Adjacent, but Distinct, Regions of Atg16L1

(A) Alignment of Atg16L1 and Atg16L2 in the WIPI2b binding region. The acidic residues in Atg16L1 (highlighted in red) that are not conserved in Atg16L2 were individually mutated to arginine (basic residues in blue, noncharged in green).

(B) Lysates from HEK293A cells transfected with GFP-WIPI2b were mixed with lysates from HEK293A cells transfected with FLAG-Atg16L2, FLAG-Atg16L1, or Flag-Atg16L1 mutants, immunoprecipitated, and analyzed by immunoblotting.

(C) Statistical analysis of FLAG-Atg16L1 binding in (B) was performed by Student's t test. SEM for n = 4. *$p < 0.05$.

(D) Cell lysates from HEK293A cells transiently expressing FLAG-Atg16L2, FLAG-Atg16L1, or FLAG-Atg16L1 mutants were subjected to immunoprecipitation using FLAG M2 agarose beads. Protein complexes were analyzed using immunoblotting.

(E) Statistical analysis of FIP200 binding in (D) was performed by Student's t test. SEM for n = 3. *$p < 0.05$.

(legend continued on next page)

bound most strongly to Atg16L1 1–230, while GFP-WIPI1a showed an increased binding to Atg16L1 1–230; neither bound to 1–207. These data suggest that the WIPI2b-binding site is located in Atg16L1 between 207 and 230.

Atg16L2, an isoform of Atg16L1, cannot function in autophagy, possibly due to its inability to localize to phagophores (Ishibashi et al., 2011). A region in Atg16L1 (229–242) was identified that is poorly conserved in Atg16L2 and may be important in membrane localization (Ishibashi et al., 2011). As this region overlaps with the WIPI2 binding region, we tested if WIPI2b binds Atg16L2. As shown in Figure 2D, GFP-WIPI2b does not bind to Atg16L2.

WIPI2 and FIP200 Bind to Independent Sites on Atg16L1

To identify residues required for WIPI2b binding, we aligned residues 207–242 of Atg16L1 and Atg16L2 (Figure 3A). The main difference between the two proteins in this region is the presence of acidic residues in Atg16L1 that are not conserved in Atg16L2. To test if these acidic residues in Atg16L1 mediated binding to WIPI2b, we separately mutated all nine residues to arginine (to mimic the charge in Atg16L2) and tested the binding of these FLAG-tagged charge-change mutants to GFP-WIPI2b (Figures 3B and 3C). Mutation of either residue E226 or E230 to arginine (E226R and E230R) abolished binding of Atg16L1 to WIPI2b, and E208R or D212R mutants reproducibly showed reduced binding.

FIP200 binds Atg16L1 in the region between 230 and 242 (Gammoh et al., 2013, Nishimura et al., 2013). This region encompasses part of the WIP2b binding site, so we asked if the WIPI2 and FIP200 binding sites overlapped by testing if our FLAG-Atg16L1 constructs could bind FIP200 (Figures 3D and 3E). Endogenous FIP200 bound to wild-type Atg16L1, but not to Atg16L2. FIP200 also bound to the point mutants that reduce or abolish WIPI2 binding (E208R, D212R, E226R, and E230R). However, the Atg16L1 point mutations from E235 to E241 bound very weakly or not at all to FIP200. These results show distinct, but adjacent, binding sites for WIPI2b and FIP200 within 207–242 of Atg16L1. Recent data suggest that the FIP200 binding site on Atg16L1 encompasses residue 246 (Fujita et al., 2013), so we extended residue mapping to E249 and saw a recovery of FIP200 binding to wild-type (WT) Atg16L1 (Figure S3A).

We next asked if the binding of WIPI2 to Atg16L1 in MEFs lacking FIP200 was altered compared to wild-type MEFs. Endogenous WIPI2b and Atg16L1 coimmunoprecipitated in both WT and FIP200$^{-/-}$ MEF cells, with no significant difference, demonstrating that FIP200 is not required for the WIPI2b-Atg16L1 interaction (Figure 3F). In support of this finding, in approximately 10% of FIP200$^{-/-}$ MEFs, WIPI2 can be found on LC3-positive tubular structures (Figure S3B). As expected, WIPI2 puncta are observed in autophagy-deficient MEFs including Atg5 and Atg3, which are LC3 negative (Figure S3B). Additionally, double charge-change mutants of Atg16L1 that cannot bind WIPI2b have unaltered binding to FIP200, and vice versa (Figure 3G). A model of Atg16L1 207–246 (Figure 3H) shows that the WIPI2b and FIP200 binding sites on Atg16L1 lie on either side of a proline-induced loop, suggesting that one surface of Atg16L1 would bind to WIPI2, while a distinct surface opposite would bind FIP200. Note that a trimeric complex between GFP-WIPI2b, FLAG-Atg16L1, and FIP200 can exist as shown in Figure 4G.

Mutational Analysis of WIPI2b Reveals the Binding Mechanism for Atg16L1

WIPI2b is a member of the WD-repeat SVP1-like family of seven-bladed β-propeller proteins that bind phosphatidylinositols (PROPPINs) (Dove et al., 2004). All known members have two PtdIns(3)P binding sites formed around a conserved FRRG motif and a hydrophobic loop that is predicted to insert into the membrane (Baskaran et al., 2012, Krick et al., 2012, Watanabe et al., 2012) (Figures S4A and S4B). Recruitment of the PtdIns(3)P effector Atg18 and its homologs (Krick et al., 2008, Obara et al., 2008, Nair et al., 2010, Polson et al., 2010) to PtdIns(3)P occurs via a conserved FRRG motif. We began mapping the Atg16L1 interaction site on WIPI2b using GFP-tagged WIPI proteins and blotting for coimmunoprecipitated Atg16L1. Inhibition of PtdIns(3)P binding through mutation of the PtdIns(3)P binding motif FRRG to FTTG (GFP-WIPI2b FTTG) had no significant effect on the ability of WIPI2b to bind Atg16L1 (Figures 4A and 4B). Atg16L1 bound to GFP-WIPI2b, but not detectably to GFP-WIPI2a (Figures 4A and 4E) or GFP-WIPI4 (Figure S4C). WIPI2a is identical to WIPI2b, except for an 18 amino acid insertion in between β1 and β2 of blade 1 of the β-propeller (Figures 4C, S4A, and S4D). Using our data and a model of WIPI2 based on the structure of a yeast ortholog, Hsv2 (Baskaran et al., 2012, Krick et al., 2012, Watanabe et al., 2012) (Figure 4C), we predicted possible sites on WIPI2 that might bind Atg16L1. As the site on Atg16L1 is acidic, we looked for conserved basically charged residues proximal to the site of the 18 amino acid insert in the WIPI2a isoform (which does not bind Atg16L1). We identified two solvent-exposed arginine residues, R108 and R125, in the cleft between blades 2 and 3 of WIPI2b (Figure 4D). Mutation of R108 to glutamate (WIPI2b R108E) reduced Atg16L1 binding dramatically, while R125E showed decreased binding (Figure 4D).

These arginine residues (R108 and R125) are well conserved in WIPI1a, but we detected only a weak binding of WIPI1a to Atg16L1 (Figures 2C and 2D). As the C-terminal domains of the PROPPINs are less well conserved (Baskaran et al., 2012, Krick et al., 2012), we tested the role of the C terminus in Atg16L1 binding. Deletion of the C terminus of WIPI2b significantly increased binding to Atg16L1 (Figures 4E and 4F), and this was mirrored by a small, but significant, increase in WIPI1a ΔCT binding to Atg16L1. These results suggest that the conserved Atg16L1-binding site is potentially masked by the insertion in WIPI2a and that the C-terminal domains may modulate binding.

To test if the WIPI2b-Atg16L1 interaction is direct, we used charge-change binding experiments with the WIPI2b and

(F) WIPI2 was immunoprecipitated from lysates from WT or FIP200$^{-/-}$ MEFS after treatment with 0.5 mM DSP. Bound Atg16 and Atg12–5 were detected by immunoblotting.
(G) HEK293 cells stably expressing GFP-WIPI2b were and transiently transfected either FLAG-Ag16L1 WT, E226R E230R (ERER), or D237R D239R (DRDR). Bound and input (bottom) were analyzed by immunoblotting.
(H) Structural model of the region of Atg16L1 207–246 that interacts with WIPI2b and FIP200. See also Figure S3 and Table S1.

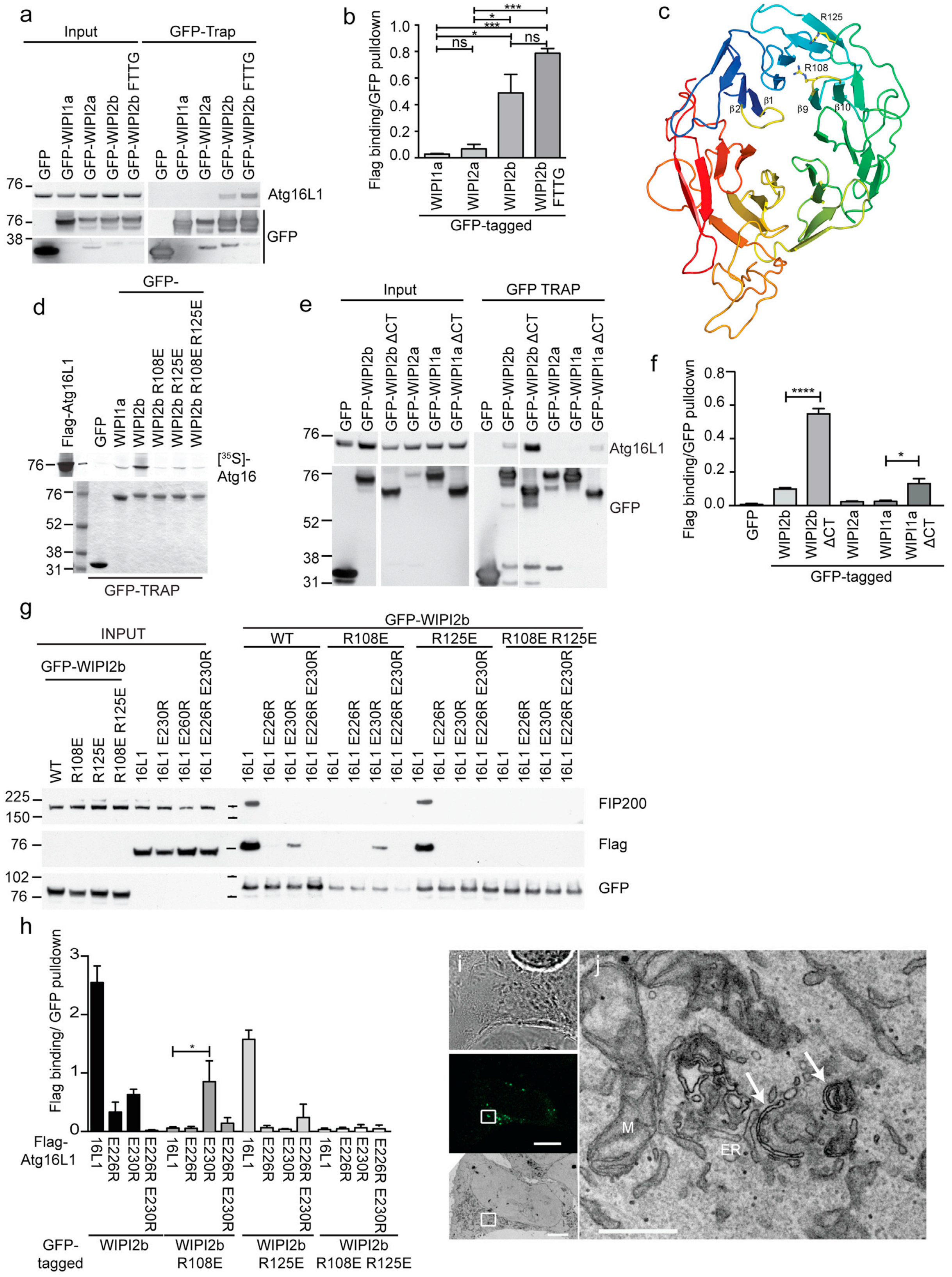

(legend on next page)

Atg16L1 mutants. We mixed cell lysates expressing WT, single, or double point mutants of GFP-WIPI2b and FLAG-Atg16L1 in all possible permutations (Figures 4G and S5A). The WIPI2b-Atg16L1 interaction is significantly restored when the GFP-WIPI2b R108E mutant is mixed with FLAG-Atg16L1 E230R, compared to FLAG-Atg16L1 binding to GFP-WIPI2b R108E (Figure 4H). This implies that residues R108 in WIPI2b and E230 in Atg16L1 interact directly. A proposed model of the predicted binding site on WIPI2b with Atg16L1 (207–246) interaction is shown in Figure S5B.

WIPI2b Binding to Atg16L1 Is Required for LC3 Localization and Autophagy

We used the double charge-change mutant GFP-WIPI2b R108E R125E (RERE) that completely abolishes Atg16L1 binding to probe the function of the WIPI2b-Atg16L1 interaction in starvation-induced autophagy. In WIPI2-depleted cells, WT GFP-WIPI2b increased starvation-induced LC3-II levels; however, expression of GFP-WIPI2b RERE inhibited autophagy (Figures 5A and 5B). This inhibition in WIPI2-depleted cells may be due to GFP-WIPI2b RERE outcompeting any residual endogenous WIPI2b for PtdIns(3)P binding and therefore stalling autophagosome formation by blocking Atg12–5-16L1 recruitment. Consistently, the inhibition of LC3 lipidation by GFP-WIPI2b RERE was dependent on the PtdIns(3)P binding ability of WIPI2b: expression of GFP-WIPI2b RERE FTTG (which is unable to bind PtdIns(3)P) did not inhibit LC3 lipidation in WIPI2-depleted cells (Figures 5C and 5D). Levels of SQSTM-1/p62, an autophagy cargo receptor, are used to monitor autophagic flux (Klionsky et al., 2012). p62 accumulated after expression of GFP-WIPI2b RERE in WIPI2-depleted cells, and this was abolished in the WIPI2b RERE FTTG mutant (Figures 5C and 5D). GFP-WIPI2b FTTG had no inhibitory effect on LC3 lipidation or p62 levels.

Immunofluorescence analysis of WIPI2-depleted HEK cells shows that WT WIPI2b and WIPI2b RERE form puncta upon autophagy initiation (Figures 5E and 5F). GFP-WIPI2b RERE puncta are open autophagosomes (Figures 4I and 4J). The PtdIns(3)P-binding deficient WIPI2b FTTG and WIPI2b RERE FTTG do not form puncta (Figures 5E and 5F). Consistent with LC3 lipidation data, WT WIPI2b puncta are LC3 positive, while the WIPI2b RERE puncta are LC3 negative, and the numbers of LC3-postive autophagosomes are reduced by expression of WIPI2b RERE, but not after expression of WIPI2b RERE FTTG (Figures 5E and 5G). These results show that WIPI2b binding to both Atg16L1 and PtdIns(3)P is necessary for LC3 lipidation and autophagosome formation.

Ectopic Localization of WIPI2b to the Plasma Membrane Is Sufficient to Drive LC3 Lipidation through Recruitment of the Atg16L1 Complex

Our data suggest that WIPI2b functions in autophagy by recruiting the Atg12–5-16L1 complex to PtdIns(3)P-positive membranes by binding Atg16L1. To confirm this, we tested if ectopic localization of WIPI2b to the plasma membrane was sufficient to drive LC3 lipidation. We used mCherry-WIPI2b-CAAX constructs based on the KRas CAAX sequence (Ahearn et al., 2012) using WIPI2b-FTTG constructs. Ectopically localized Atg16L1 can recruit LC3 to the plasma membrane (Fujita et al., 2008). Expression of mCherry-WIPI2b-CAAX significantly increased LC3-II in fed and wortmannin-treated starved cells (Figures 6A and 6B), and this increase was lost in mCherry-WIPI2b-RERE-CAAX mutants unable to bind Atg16L1 (Figures 6A–6C). Both mCherry-WIPI2b-CAAX and mCherry-WIPI2b-RERE-CAAX were plasma membrane localized, but GFP-LC3 was only recruited by mCherry-WIPI2b-CAAX, and this occurred in fed, starved, or wortmannin-treated 2GL9 cells (Figure 6D) or MCF-7 cells (Figure S6). We conclude that WIPI2b membrane localization is sufficient to recruit the Atg12–5-16L1 complex and subsequently drive LC3 lipidation independently of upstream signals, including PI3-kinases and mTORC1 inactivation.

As WIPI2b-CAAX can drive LC3 lipidation and recruitment to the plasma membrane, we asked if double-membrane phagophores could be detected on or emerging from the plasma membrane. In the regions on the plasma membrane that contain mCherry-WIPI2b-CAAX and GFP-LC3, no typical double membranes were detected in the cells examined, but in all doubly transfected cells, we saw a cluster of small vesicular structures under the plasma membrane (Figures 6E and 6F). The nature of these vesicles requires characterization.

Figure 4. WIPI2 Binds Atg16L1 though R108E and R125E in a Solute-Exposed Cleft in WIPI2

(A) Lysates from HEK293A cells transiently expressing GFP, GFP-WIPI1a, GFP-WIPI2a, GFP-WIPI2b, or GFP-WIPI2b FTTG mutant were used for GFP-Trap. Endogenous Atg16L1 binding was analyzed by immunoblot.

(B) Statistical analysis of (A) was performed by one-way ANOVA with Tukey's posttest. SEM for $n = 3$. $^{*}p < 0.05$.

(C) Model of WIPI2b based on crystal structure of *Kluyveromyces marxianus* Atg18 (Protein Data Bank ID 3VU4) (Watanabe et al., 2012). The $\beta1$-$\beta2$ loop colored yellow is the site of an18 aa insertion in WIPI2a.

(D) GFP-Trap from HEK293A cells transiently expressing GFP, GFP-WIPI1a, GFP-WIPI2b, GFP-WIPI2b R108E, GFP-WIPI2b R125E, or GFP-WIPI2b R108E R125E was mixed with in vitro translated ^{35}S-labeled FLAG-Atg16L1 and analyzed by autoradiography.

(E) Lysates from HEK293A cells transiently expressing GFP, GFP-WIPI2b, GFP-WIPI2b ΔCT, GFP-WIPI2a, GFP-WIPI1a, or GFP-WIPI1a ΔCT were used for GFP-Trap. Endogenous Atg16L1 binding was analyzed by immunoblot.

(F) Statistical analysis of (E) was performed by one-way ANOVA with Tukey's posttest. SEM for $n = 2$. $^{*}p < 0.05$.

(G) Lysates from HEK293A cells transiently expressing GFP-WIPI2b WT, GFP-WIPI2b R108E, R125E, or R108E R125E were mixed with lysates from HEK293A cells transiently expressing FLAG-Atg16L1 WT, E226R, E230R, or E226R E230R in all possible permutations. Protein complexes from mixed lysates were immunoprecipitated using GFP-Trap, followed by immunoblot analysis.

(H) Statistical analysis of (G) was performed by one-way ANOVA with Tukey's posttest. SEM from $n = 3$. $^{*}p < 0.05$.

(I) CLEM analysis of HEK293 cells treated with siRNA to WIPI2 were transfected with GFP-WIPI2b RERE and starved in EBSS. Bright-field image (top), GFP-WIPI2 RERE signal (middle), TEM of selected cell (bottom). Boxed area shown in (J). Scale bars represent 10 μM for bright-field and confocal and 5 μM for TEM.

(J) High-magnification TEM of boxed area in (I). Arrows indicate open phagophores in the vicinity of ER and mitochondria (M), which contain GFP-WIPI2b RERE.

See also Figures S4 and S5 and Table S1.

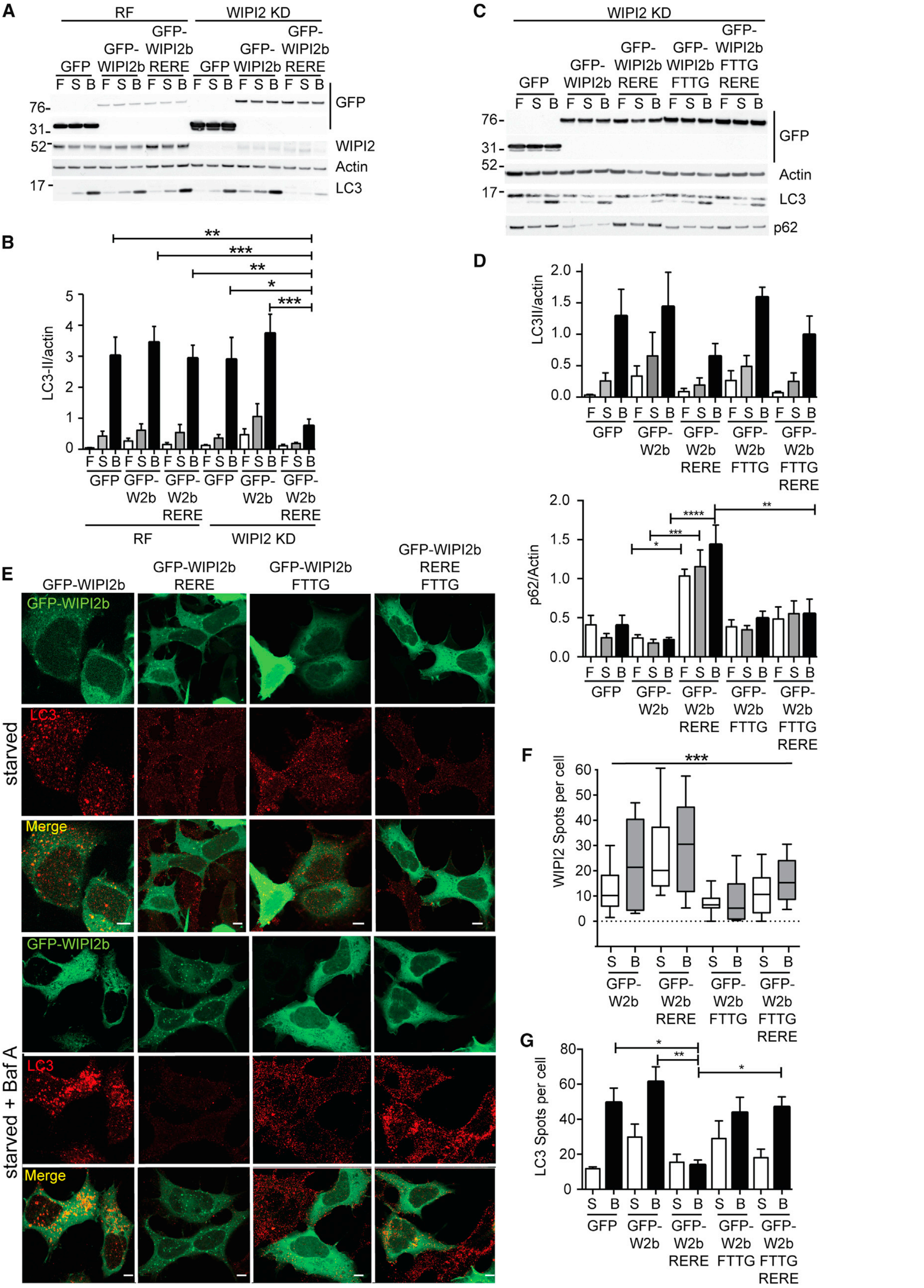

(legend on next page)

To determine whether WIPI2b-CAAX-driven lipidation requires FIP200, we expressed hemagglutinin (HA)-WIPI2b-CAAX (FTTG) in RISC-free control, ATG16L1, or FIP200 siRNA-treated cells. CAAX-driven LC3 lipidation was lost in Atg16L1-depleted cells, but not in FIP200-depleted cells (Figure 6G), demonstrating that LC3 recruitment to the plasma membrane via WIPI2b-CAAX requires Atg16L1, but not FIP200.

WIPI2b, but Not FIP200, Binding Is Required for Atg16L1 to Rescue Autophagy in Atg16L1$^{\Delta/\Delta}$ MEFs

Atg16L1$^{\Delta/\Delta}$ MEFs do not support starvation-induced LC3 lipidation or autophagy, as they contain Atg16L1 with a deletion (Δ69–213) (Saitoh et al., 2008). To test the requirement for WIPI2b and FIP200 binding to Atg16L1 for autophagy, we expressed Atg16L1 in Atg16L1$^{\Delta/\Delta}$ MEFs. We used WT or Atg16L1 mutants of either the WIPI2b-binding site (E226R E239R/ERER) or the FIP200-binding site (D237R D239R/DRDR) to abolish WIPI2b or FIP200 binding, respectively (see Figure 3G). The number of LC3-positive autophagosomes formed per cell was significantly less with FLAG-Atg16L1 ERER mutant, which cannot bind WIPI2b, compared to WT rescue; in addition, LC3 lipidation was not rescued by this mutant (Figures 7A–7D). In contrast, there was no significant difference between the number of autophagosomes with WT FLAG-Atg16L1 and FLAG-Atg16L1 DRDR, and there was a significant restoration of LC3 lipidation (Figures 7A–7D). Clearly, Atg16L1 must bind WIPI2b to function in autophagy. Additionally, Atg16L1 mutants that cannot bind FIP200, but can bind WIPI2, rescue autophagy to an extent similar to that of WT Atg16L1.

WIPI2b Is Required for LC3 Recruitment during *Salmonella* Infection

Intracellular bacterial pathogens, such as *Salmonella*, are targeted by autophagy as part of a host cell bacterial clearance response (Birmingham et al., 2006). After invasion, *Salmonella* resides in a specialized organelle, the *Salmonella*-containing vacuole (SCV) (Gorvel and Méresse, 2001). Phagophore membranes form around the bacteria following *Salmonella*-induced damage to the SCV membrane (Fujita et al., 2013, Birmingham et al., 2006, Kageyama et al., 2011). As in canonical autophagy, LC3 is conjugated to these phagophore membranes using Atg12–5-16L1 (Kageyama et al., 2011); however, recruitment of the Atg12–5-16L1 complex involves a number of redundant mechanisms (Fujita et al., 2013). Both FIP200 and ubiquitin binding by Atg16L1 are important for LC3 recruitment (Fujita et al., 2013). After infection with *Salmonella*, WIPI2 on p62-positive SCVs colocalized with LC3 (Figure 7E), LAMP1, and ubiquitin (Figures S7A and S7B). These WIPI2-positive *Salmonella* were detected on ER structures (Figure S7C) as expected (Huang et al., 2011). We asked if WIPI2 interaction with ATG16L1 was also required for the recruitment of the LC3 to the phagophore during bacterial infection. WIPI2 depletion significantly reduced LC3 on the SCV (Figures 7E and 7F). Autophagy restricts bacterial proliferation; we therefore assayed for replication in HeLa cells treated with either control or WIPI2 siRNA (Figure S7D). Colony formation was significantly increased in cells lacking WIPI2. To differentiate between the FIP200 and WIPI2 binding requirements for Atg12–5-16L1 recruitment to the phagophore during *Salmonella* infection, we transfected Atg16L1$^{\Delta/\Delta}$ MEFs with WT FLAG-Atg16L1, ERER (WIPI2-binding mutant), or DRDR (FIP200-binding mutant) (Figure 7G). Significantly less LC3 was recruited to p62-positive *Salmonella* in cells expressing the WIPI2-binding mutant compared to WT Atg16L1 (Figure 7H). There was no significant difference between rescue with the FIP200-binding mutant and WT Atg16L1. We conclude that WIPI2 is required for Atg12–5-16L1 complex recruitment and subsequent LC3 lipidation during autophagic targeting of *Salmonella*.

DISCUSSION

Here, we show that the PtdIns(3)P binding protein WIPI2b directly binds Atg16L1 and recruits the Atg12–5-16L1 complex during starvation-induced autophagy and during clearance of pathogenic intracellular bacteria. The Atg16L1 complex acts as an E3-like enzyme for LC3 lipidation, an essential step in autophagosome formation, and the mechanism of its recruitment to membranes has been an unresolved question.

We propose that WIPI proteins recruit upstream effectors to the site of phagophore formation, the omegasome, in a PtdIns(3)P-dependent manner. There are four members of the WIPI family of proteins, WIPI1–WIPI4. These proteins are PROPPINs, β-propeller-containing proteins that bind PtdIns (Michell et al., 2006). WIPI homologs, including the Atg18 proteins in *S. cerevisiae* (Barth et al., 2001, Guan et al., 2001), *S. pombe* (Sun et al., 2013), and the *C. elegans* WIPI4 homolog Epg-6, which is downstream of the *C. elegans* Atg18 (WIPI1/2 homolog) (Lu et al., 2011), have the ability to bind PtdIns(3)P but have different interactors. *S. cerevisiae* Atg18 (Rieter et al., 2013) and Epg-6 (Lu et al., 2011) interact with Atg2, as does WIPI4 (Velikkakath et al., 2012, Lu et al., 2011). Yeast Atg16 proteins do not have the C terminus containing the WIPI2b binding sites.

Figure 5. WIPI2 Function in Amino Acid Starvation Requires Atg16L1 and PI3P Binding

(A) GFP, siRNA-resistant GFP-WIPI2b, or GFP-WIPI2b RERE was expressed in HEK293A cells treated for 72 hr with either RISC-free (RF) or WIPI2 siRNA. Cells were left in full medium (F) or starved for 2 hr with EBSS (S) or EBSS with BafA (B) before immunoblot analysis.

(B) Statistical analysis of (A). SEM for n = 3. Statistical analysis was performed by one-way ANOVA with Tukey's posttest. *$p < 0.05$.

(C) GFP, siRNA-resistant GFP-WIPI2b, GFP-WIPI2b RERE, GFP-WIPI2b FTTG, or GFP-WIPI2b FTTG RERE was expressed in HEK293A cells treated for 72 hr with WIPI2 siRNA. Cells were left in full medium (F) or starved for 2 hr with EBSS (S) or EBSS with BafA (B) before immunoblot analysis.

(D) Statistical analysis of (C) was performed by one-way ANOVA with Tukey's posttest. The SEM for LC3 (n = 2) and p62 (n = 4) are shown. *$p < 0.05$.

(E) siRNA-resistant GFP-WIPI2b, GFP-WIPI2b RERE, GFP-WIPI2b FTTG, or GFP-WIPI2b FTTG RERE was expressed in HEK293A cells treated for 72 hr with WIPI2 siRNA. Cells were starved in EBSS for 2 hr without or with BafA, fixed, and labeled, and LC3 was visualized by confocal microscopy. Scale bars, 10 μm.

(F) Quantification of WIPI2 puncta per cell from (E). SEM from 10 cells per condition; ***$p < 0.001$ using one-way ANOVA.

(G) Statistical analysis of LC3 puncta from (E) with GFP control (not shown in E). SEM for n = 3. Statistical analysis was performed by one-way ANOVA with Dunn's posttest t test. *$p < 0.05$. See also Table S1.

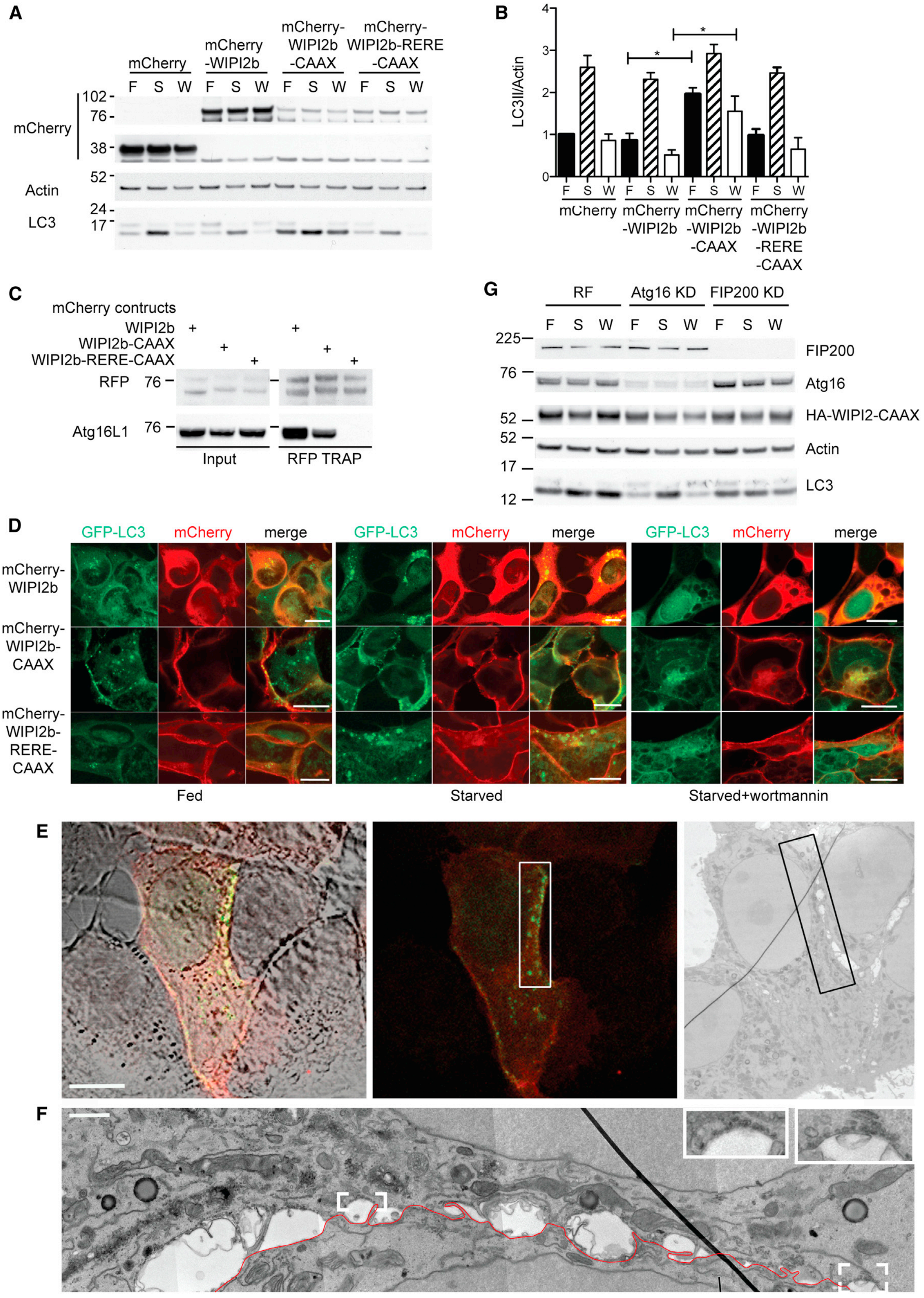

(legend on next page)

However, *S. pombe* Atg18 a binds Atg5, and this binding is required for the targeting of the Atg12–5-16 complex to the PAS (Sun et al., 2013), implying that the overall function of Atg18/WIPI2 in Atg16 complex recruitment may be conserved between *S. pombe* and human.

In mammals, WIPI2 and WIPI4 have different functions (Lu et al., 2011, Polson et al., 2010, Velikkakath et al., 2012). We show that WIPI2b binds and recruits Atg12–5-16L1, and we speculate, based on the data from *C. elegans*, that WIPI4 may act after this step and recruit Atg2 similarly to *S. cerevisiae* Atg18 (Rieter et al., 2013), but not Atg12–5-16. Atg2 has been shown to bind WIPI4 and is essential for autophagy and lipid droplet formation (Velikkakath et al., 2012).

Whether the WIPI2-Atg16L1 interaction is regulated is not clear. Although the interaction is starvation independent (see Figure 1A), the formation of WIPI2-Atg16L1-positive puncta requires starvation. Intriguingly, WIPI1a, which contains the two conserved Atg16L1-binding residues, shows a very weak binding to Atg16L1 unless a truncated form of Atg16L1 (1–230) or a C-terminal deletion of WIPI1a is used. This suggests that the binding between the WIPI2a and Atg16L1 may be limited by its C terminus and Atg16L1 residues between 230 and 242.

Recruitment of LC3 to *Salmonella* requires the Atg12–5-16L1 complex (Birmingham et al., 2006, Kageyama et al., 2011), and the requirement for PtdIns(3)P-binding proteins in this process is unclear (Huang et al., 2011, Kageyama et al., 2011). However, the targeting of Atg12–5-16L1, and subsequently LC3, to bacteria appears to be complex, with redundancy present in the system (Fujita et al., 2013). Therefore, subtle effects of removing one of the Atg12–5-16L1 recruitment mechanisms may be difficult to observe, as in the recent identification of ubiquitin binding by Atg16L1 and FIP200 (Fujita et al., 2013). Our results show that WIPI2b is also required for targeting of the *Salmonella* SCV by the autophagic machinery.

Autophagy is an ancient cellular process found from yeast to humans. For example, the primitive metazoan *Hydra* undergoes starvation-induced autophagy, and omegasomes are seen in *C. elegans*. Although the core components in metazoans are evolutionarily conserved, they are distinct from yeast. The WIPI2b and Atg16L1 interaction sites are conserved in starlet sea anemone, and the WIPI2b site is conserved in the ancient marine sponge *Amphimedon*, indicating that this mechanism of recruitment of the LC3 conjugation machinery via WIPI2b emerged early in metazoan evolution. It is likely that the WIPI2b-Atg16L1 interaction occurs in most metazoan groups. Interestingly, the FIP200-binding site is missing in metazoans predating emergence of zebrafish and may only be present in vertebrates. The Atg16L1-binding site of WIPI2b is conserved in the *S. cerevisiae* homolog Atg18, although the WIPI2b-binding site in Atg16L1 is not present in yeast Atg16.

The binding sites for WIPI2b and FIP200 are adjacent on Atg16L1, and we show that WIPI2 and FIP200 can bind Atg16L1 both individually and simultaneously. However, we did not observe interaction between WIPI2 and FIP200 after overexpression of ATG16L1 (data not shown). FIP200, a component of the ULK complex that regulates autophagy activation, was shown to recruit Atg16L1 (Gammoh et al., 2013, Nishimura et al., 2013). However, our mapping experiments show that the mutations used previously will have abolished WIPI2b binding, and therefore the inability of these FIP200 constructs to rescue autophagy may be attributable to the loss of the WIPI2-Atg16L1 interaction. An Atg16L1 mutant that cannot bind WIPI2, but can bind FIP2000, is unable to rescue autophagy, suggesting that WIPI2b rather than FIP200 is responsible for Atg16L1 recruitment. The close proximity of the WIPI2b and FIP200 binding sites on Atg16L1 is intriguing, and there is potential for regulation or cooperation between WIPI2b and FIP200 at this site.

FIP200 can bind to Atg16L1 Δ78–230 (Gammoh et al., 2013, Nishimura et al., 2013) that lacks the coiled-coil domain required for dimerization, suggesting that the affinity of monomeric Atg16L1 for FIP200 is strong enough to make a stable interaction. However, the human Atg16L1 ΔCCD (70–212) construct, also lacking the coiled-coil domain, did not bind WIPI2 (Figure S3B). This suggests that Atg16L1 monomers have a weak affinity for WIPI2 and that dimerization is required for Atg16L1 to be recruited to membranes by WIPI2b. We modeled the interaction between WIPI2b and Atg16L1 based on the crystal structures of Hsv2 and a model of Atg16L1 (207–265), taking into account the residues required for complex formation (Figure S5B). This model suggests that an Atg16L1 dimer would be perpendicular to the membrane. We propose that WIPI2 binds PtdIns(3)P on the omegasome, and that the recruitment and positioning of the Atg12–5-16L1 complex on the membrane is critical for progression of the phagophore formation at the omegasome. Phagophores that form in close proximity to the ER are sandwiched between two cisternae of ER (Hayashi-Nishino et al., 2009, Ylä

Figure 6. Plasma Membrane Localized WIPI2b Promotes LC3 Lipidation through Binding Atg16L1 Independently of FIP200

(A) HEK293A cells transiently expressing the indicated mCherry constructs were treated in either full medium (F), starvation medium (S), or starvation medium with wortmannin (W) for 2 hr before immunoblot analysis.

(B) Statistical analysis of (A) was performed by one-way ANOVA with Tukey's post hoc test. SEM for n = 3. *$p < 0.05$.

(C) Complexes from HEK293A cells transiently expressing the indicated mCherry constructs were immunoprecipitated using RFP-Trap before analysis by immunoblotting.

(D) 2GL9 cells transiently expressing the indicated mCherry constructs treated as in (A) were fixed and visualized by confocal microscopy.

(E) CLEM of MCF7 cells expressing GFP-LC3 and mCherry-WIPI2b CAAX. Bright-field and confocal image merged (left), confocal of expressing cell (middle), low-magnification TEM of cell of interest (right). Boxed area indicates plasma membrane region showing colocalization of GFP-LC3 and mCherry-WIPI2b. Scale bar, 10 μm (n = 3).

(F) High-magnification TEM showing plasma membrane region boxed in (E). Magnified insets, in top right, show small vesicular clusters under the plasma membrane detected in mCherry-WIPI2b CAAX, GFP-LC3 expressing cells but not in untransfected cells from a control experiment.

(G) HEK293A cells treated with either RISC-free (RF), Atg16L1 siRNA, or FIP200 siRNA for 72 hr before transfection with HA-WIPI2b-CAAX were incubated in full medium (F), EBSS (S), or EBSS with wortmannin (W) for 2 hr before immunoblot analysis. Please note that all WIPI2 constructs are FTTG mutants. See also Figure S6 and Table S1.

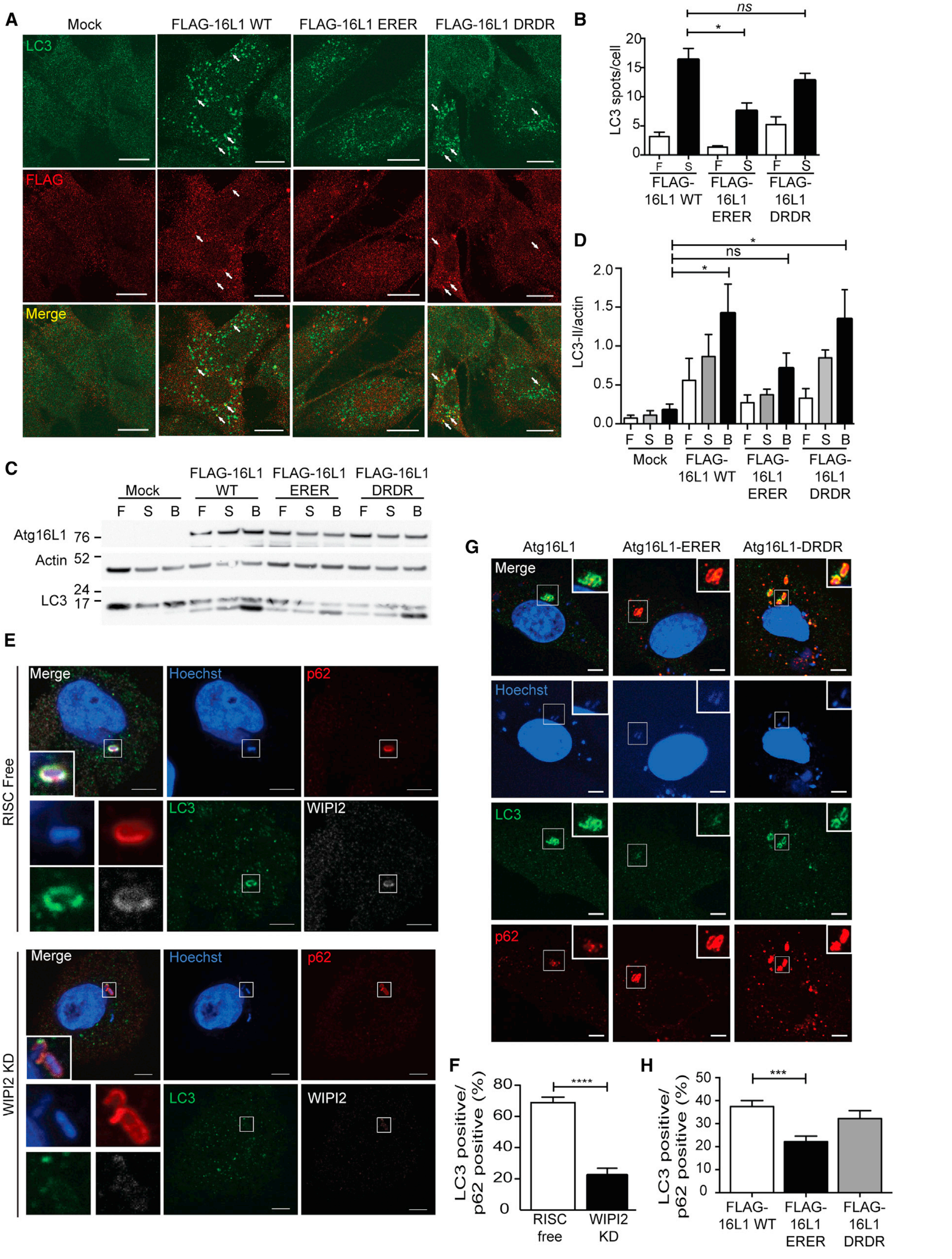

(legend on next page)

-Anttila et al., 2009), but LC3 lipidation is limited to the phagophore and does not extend onto the ER (Karanasios et al., 2013). We postulate that WIPI2 on the ER at the omegasome would position the LC3 lipidation machinery (Atg12–5-16L1 and Atg3-LC3) pointing out from the ER membrane toward the phagophore, allowing for LC3 lipidation on the phagophore membrane only. Finally, given the apparent utility of this mechanism for localizing the LC3 conjugation machinery via the WIPI2-Atg16L1 interaction, the question follows as to how positioning of this complex on a membrane gives rise in a double-membrane phagophore.

EXPERIMENTAL PROCEDURES

Cell Culture and Reagents

Cell lines, transfection protocols, siRNA, DNA constructs, primers, antibodies, and siRNA are detailed in the Supplemental Experimental Procedures.

Protein Complex Purification and Mass Spectroscopy

Protein complexes were pulled down from either fed cells or cells starved for 1 hr in Earle's balanced salt solution (EBSS) via a GFP tag using GFP-Trap beads (ChromoTek). Proteins were resolved by SDS-PAGE, fixed, and stained with GelCode, and full lanes were cut into 48 mm × 1 mm slices for tryptic digestion and mass spectrometry analysis. For more details, please see Supplemental Experimental Procedures.

Immunoprecipitation

Endogenous immunoprecipitation was performed using DSP (Lomant's Reagent) (Thermo Scientific) on intact cells at the indicated concentrations for 30 min on ice. Tagged proteins were immunoprecipitated with either GFP-Trap beads or anti-FLAG M2 affinity gel (Sigma). See Supplemental Experimental Procedures for more information.

In Vitro Translation

In vitro translated protein was produced using TNT Quick Coupled Transcription/Translation System (Promega) and EasyTag [^{35}S]-Methonine (PerkinElmer).

Bacterial Infection

Salmonella Typhimurium SL 1344, a kind gift from David Holden, was grown in Luria broth (LB) overnight. Overnight cultures were diluted 100× in LB and grown to mid-to-late log phase at optical density 600 (OD_{600}) = 2.0–2.5. Mammalian cell cultures in antibiotic-free Dulbecco's modified Eagle's medium (DMEM) were infected at a multiplicity of infection (moi) of 100 (HeLa) or 25 (MEF cell lines), centrifuged (2000 × *g* for 10 min at room temperature), and incubated at 37°C for 20 min. After 20 min, the medium was replaced with fresh medium containing gentamicin (50 μg/ml) and incubated at 37°C for 1 hr. Cells were then washed with PBS and fixed for immunofluorescence. Colony-forming unit assays were performed as described (Tattoli et al., 2012).

SUPPLEMENTAL INFORMATION

Supplemental Information includes Supplemental Experimental Procedures, a list of primers used in this study, seven figures, and one movie and can be found with this article online at http://dx.doi.org/10.1016/j.molcel.2014.05.021.

ACKNOWLEDGMENTS

H.C.D., M.R., H.E.J.P., and S.A.T. are funded by Cancer Research UK, M.I.W. is funded by BBSRC, and S.E.G. is funded by Burroughs Wellcome Fund and the CIHR. We thank the members of the lab for comments and suggestions and Chris Lamb for critical reading of the manuscript. We are grateful to colleagues N. Mizushima, M. Komatsu, S. Akira, and J.L. Guan for the MEF cell lines used in this study: We thank T. Yoshimori for the Atg16L1 constructs and M. Fukuda for Atg16L2. We thank D. Holden for the *Salmonella* Typhimurium.

Received: January 29, 2014
Revised: April 16, 2014
Accepted: May 14, 2014
Published: June 19, 2014

REFERENCES

Ahearn, I.M., Haigis, K., Bar-Sagi, D., and Philips, M.R. (2012). Regulating the regulator: post-translational modification of RAS. Nat. Rev. Mol. Cell Biol. *13*, 39–51.

Axe, E.L., Walker, S.A., Manifava, M., Chandra, P., Roderick, H.L., Habermann, A., Griffiths, G., and Ktistakis, N.T. (2008). Autophagosome formation from membrane compartments enriched in phosphatidylinositol 3-phosphate and dynamically connected to the endoplasmic reticulum. J. Cell Biol. *182*, 685–701.

Barth, H., Meiling-Wesse, K., Epple, U.D., and Thumm, M. (2001). Autophagy and the cytoplasm to vacuole targeting pathway both require Aut10p. FEBS Lett. *508*, 23–28.

Baskaran, S., Ragusa, M.J., Boura, E., and Hurley, J.H. (2012). Two-site recognition of phosphatidylinositol 3-phosphate by PROPPINs in autophagy. Mol. Cell *47*, 339–348.

Birmingham, C.L., Smith, A.C., Bakowski, M.A., Yoshimori, T., and Brumell, J.H. (2006). Autophagy controls Salmonella infection in response to damage to the Salmonella-containing vacuole. J. Biol. Chem. *281*, 11374–11383.

Chan, E.Y.W., Kir, S., and Tooze, S.A. (2007). siRNA screening of the kinome identifies ULK1 as a multidomain modulator of autophagy. J. Biol. Chem. *282*, 25464–25474.

Choi, A.M.K., Ryter, S.W., and Levine, B. (2013). Autophagy in human health and disease. N. Engl. J. Med. *368*, 651–662.

Dove, S.K., Piper, R.C., McEwen, R.K., Yu, J.W., King, M.C., Hughes, D.C., Thuring, J., Holmes, A.B., Cooke, F.T., Michell, R.H., et al. (2004). Svp1p defines a family of phosphatidylinositol 3,5-bisphosphate effectors. EMBO J. *23*, 1922–1933.

Figure 7. Atg16L1 Mutants Unable to Bind WIPI2 Cannot Rescue LC3 Lipidation or LC3 Recruitment to *Salmonella* in Atg16L1$^{\Delta/\Delta}$ MEFs

(A) Atg16L1$^{\Delta/\Delta}$ MEFs were transiently transfected with either mock, FLAG-tagged Atg16L1 WT, ERER, or DRDR. After 2 hr in EBSS, cells were fixed and labeled with anti-LC3 and anti-FLAG antibodies and visualized by confocal microscopy. Scale bars, 20 μm.
(B) Statistical analysis of (A) was performed using one-way ANOVA with Dunn's posttest. *$p < 0.05$. SEM for n = 3.
(C) Atg16L1$^{\Delta/\Delta}$ MEFs were transiently transfected with either mock, FLAG-tagged Atg16L1 WT, E226R E230R (ERER), or D237R D239R (DRDR) before analysis by immunoblotting.
(D) Statistical analysis of (C) was performed by one-way ANOVA with Tukey's post hoc test. SEM for n = 3. *$p < 0.05$.
(E) HEK293A cells were treated with either RISC-free control or WIPI2 siRNA before infection with *Salmonella* (moi = 100) for 1 hr, labeled with anti-LC3, WIPI2, and p62 antibodies, and followed by confocal analysis. Scale bars, 5 μm.
(F) Statistical analysis for (E) was performed using an unpaired Student's t test. *$p < 0.05$. SEM for n = 3.
(G) Atg16L1$^{\Delta/\Delta}$ MEFs were transiently transfected with FLAG-Atg16L1 WT, FLAG-Atg16L1 ERER, or FLAG-Atg16L2 DRDR 24 hr before being infected with *Salmonella* (moi = 25) for 1 hr, labeled with anti-LC3, anti-p62, and anti-FLAG antibodies, and followed by analysis by confocal microscopy. Scale bars, 5 μm.
(H) Statistical analysis of (G) was performed by one-way ANOVA with Dunn's post hoc test. SEM for n = 4. *$p < 0.05$. See also Figure S7.

Fujita, N., Itoh, T., Omori, H., Fukuda, M., Noda, T., and Yoshimori, T. (2008). The Atg16L complex specifies the site of LC3 lipidation for membrane biogenesis in autophagy. Mol. Biol. Cell *19*, 2092–2100.

Fujita, N., Morita, E., Itoh, T., Tanaka, A., Nakaoka, M., Osada, Y., Umemoto, T., Saitoh, T., Nakatogawa, H., Kobayashi, S., et al. (2013). Recruitment of the autophagic machinery to endosomes during infection is mediated by ubiquitin. J. Cell Biol. *203*, 115–128.

Gammoh, N., Florey, O., Overholtzer, M., and Jiang, X. (2013). Interaction between FIP200 and ATG16L1 distinguishes ULK1 complex-dependent and -independent autophagy. Nat. Struct. Mol. Biol. *20*, 144–149.

Gorvel, J.-P., and Méresse, S. (2001). Maturation steps of the Salmonella-containing vacuole. Microbes Infect. *3*, 1299–1303.

Guan, J., Stromhaug, P.E., George, M.D., Habibzadegah-Tari, P., Bevan, A., Dunn, W.A., Jr., and Klionsky, D.J. (2001). Cvt18/Gsa12 is required for cytoplasm-to-vacuole transport, pexophagy, and autophagy in Saccharomyces cerevisiae and Pichia pastoris. Mol. Biol. Cell *12*, 3821–3838.

Hayashi-Nishino, M., Fujita, N., Noda, T., Yamaguchi, A., Yoshimori, T., and Yamamoto, A. (2009). A subdomain of the endoplasmic reticulum forms a cradle for autophagosome formation. Nat. Cell Biol. *11*, 1433–1437.

Huang, J., Birmingham, C.L., Shahnazari, S., Shiu, J., Zheng, Y.T., Smith, A.C., Campellone, K.G., Heo, W.D., Gruenheid, S., Meyer, T., et al. (2011). Antibacterial autophagy occurs at PI(3)P-enriched domains of the endoplasmic reticulum and requires Rab1 GTPase. Autophagy *7*, 17–26.

Ishibashi, K., Fujita, N., Kanno, E., Omori, H., Yoshimori, T., Itoh, T., and Fukuda, M. (2011). Atg16L2, a novel isoform of mammalian Atg16L that is not essential for canonical autophagy despite forming an Atg12–5-16L2 complex. Autophagy *7*, 1500–1513.

Itakura, E., and Mizushima, N. (2010). Characterization of autophagosome formation site by a hierarchical analysis of mammalian Atg proteins. Autophagy *6*, 764–776.

Itoh, T., Fujita, N., Kanno, E., Yamamoto, A., Yoshimori, T., and Fukuda, M. (2008). Golgi-resident small GTPase Rab33B interacts with Atg16L and modulates autophagosome formation. Mol. Biol. Cell *19*, 2916–2925.

Kageyama, S., Omori, H., Saitoh, T., Sone, T., Guan, J.L., Akira, S., Imamoto, F., Noda, T., and Yoshimori, T. (2011). The LC3 recruitment mechanism is separate from Atg9L1-dependent membrane formation in the autophagic response against Salmonella. Mol. Biol. Cell *22*, 2290–2300.

Karanasios, E., Stapleton, E., Manifava, M., Kaizuka, T., Mizushima, N., Walker, S.A., and Ktistakis, N.T. (2013). Dynamic association of the ULK1 complex with omegasomes during autophagy induction. J. Cell Sci. *126*, 5224–5238.

Klionsky, D.J., Abdalla, F.C., Abeliovich, H., Abraham, R.T., Acevedo-Arozena, A., Adeli, K., Agholme, L., Agnello, M., Agostinis, P., Aguirre-Ghiso, J.A., et al. (2012). Guidelines for the use and interpretation of assays for monitoring autophagy. Autophagy *8*, 445–544.

Koyama-Honda, I., Itakura, E., Fujiwara, T.K., and Mizushima, N. (2013). Temporal analysis of recruitment of mammalian ATG proteins to the autophagosome formation site. Autophagy *9*, 1491–1499.

Krick, R., Henke, S., Tolstrup, J., and Thumm, M. (2008). Dissecting the localization and function of Atg18, Atg21 and Ygr223c. Autophagy *4*, 896–910.

Krick, R., Busse, R.A., Scacioc, A., Stephan, M., Janshoff, A., Thumm, M., and Kühnel, K. (2012). Structural and functional characterization of the two phosphoinositide binding sites of PROPPINs, a β-propeller protein family. Proc. Natl. Acad. Sci. USA *109*, E2042–E2049.

Lamb, C.A., Yoshimori, T., and Tooze, S.A. (2013). The autophagosome: origins unknown, biogenesis complex. Nat. Rev. Mol. Cell Biol. *14*, 759–774.

Lu, Q., Yang, P., Huang, X., Hu, W., Guo, B., Wu, F., Lin, L., Kovács, A.L., Yu, L., and Zhang, H. (2011). The WD40 repeat PtdIns(3)P-binding protein EPG-6 regulates progression of omegasomes to autophagosomes. Dev. Cell *21*, 343–357.

Mauthe, M., Jacob, A., Freiberger, S., Hentschel, K., Stierhof, Y.-D., Codogno, P., and Proikas-Cezanne, T. (2011). Resveratrol-mediated autophagy requires WIPI-1-regulated LC3 lipidation in the absence of induced phagophore formation. Autophagy *7*, 1448–1461.

Michell, R.H., Heath, V.L., Lemmon, M.A., and Dove, S.K. (2006). Phosphatidylinositol 3,5-bisphosphate: metabolism and cellular functions. Trends Biochem. Sci. *31*, 52–63.

Mizushima, N., Kuma, A., Kobayashi, Y., Yamamoto, A., Matsubae, M., Takao, T., Natsume, T., Ohsumi, Y., and Yoshimori, T. (2003). Mouse Apg16L, a novel WD-repeat protein, targets to the autophagic isolation membrane with the Apg12-Apg5 conjugate. J. Cell Sci. *116*, 1679–1688.

Mizushima, N., Yoshimori, T., and Ohsumi, Y. (2011). The role of Atg proteins in autophagosome formation. Annu. Rev. Cell Dev. Biol. *27*, 107–132.

Nair, U., Cao, Y., Xie, Z., and Klionsky, D.J. (2010). Roles of the lipid-binding motifs of Atg18 and Atg21 in the cytoplasm to vacuole targeting pathway and autophagy. J. Biol. Chem. *285*, 11476–11488.

Nishimura, T., Kaizuka, T., Cadwell, K., Sahani, M.H., Saitoh, T., Akira, S., Virgin, H.W., and Mizushima, N. (2013). FIP200 regulates targeting of Atg16L1 to the isolation membrane. EMBO Rep. *14*, 284–291.

Obara, K., Sekito, T., Niimi, K., and Ohsumi, Y. (2008). The Atg18-Atg2 complex is recruited to autophagic membranes via phosphatidylinositol 3-phosphate and exerts an essential function. J. Biol. Chem. *283*, 23972–23980.

Polson, H.E.J., de Lartigue, J., Rigden, D.J., Reedijk, M., Urbé, S., Clague, M.J., and Tooze, S.A. (2010). Mammalian Atg18 (WIPI2) localizes to omegasome-anchored phagophores and positively regulates LC3 lipidation. Autophagy *6*, 506–522.

Proikas-Cezanne, T., Waddell, S., Gaugel, A., Frickey, T., Lupas, A., and Nordheim, A. (2004). WIPI-1alpha (WIPI49), a member of the novel 7-bladed WIPI protein family, is aberrantly expressed in human cancer and is linked to starvation-induced autophagy. Oncogene *23*, 9314–9325.

Rieter, E., Vinke, F., Bakula, D., Cebollero, E., Ungermann, C., Proikas-Cezanne, T., and Reggiori, F. (2013). Atg18 function in autophagy is regulated by specific sites within its β-propeller. J. Cell Sci. *126*, 593–604.

Saitoh, T., Fujita, N., Jang, M.H., Uematsu, S., Yang, B.G., Satoh, T., Omori, H., Noda, T., Yamamoto, N., Komatsu, M., et al. (2008). Loss of the autophagy protein Atg16L1 enhances endotoxin-induced IL-1beta production. Nature *456*, 264–268.

Sakoh-Nakatogawa, M., Matoba, K., Asai, E., Kirisako, H., Ishii, J., Noda, N.N., Inagaki, F., Nakatogawa, H., and Ohsumi, Y. (2013). Atg12-Atg5 conjugate enhances E2 activity of Atg3 by rearranging its catalytic site. Nat. Struct. Mol. Biol. *20*, 433–439.

Slobodkin, M.R., and Elazar, Z. (2013). The Atg8 family: multifunctional ubiquitin-like key regulators of autophagy. Essays Biochem. *55*, 51–64.

Sun, L.L., Li, M., Suo, F., Liu, X.M., Shen, E.Z., Yang, B., Dong, M.Q., He, W.Z., and Du, L.L. (2013). Global analysis of fission yeast mating genes reveals new autophagy factors. PLoS Genet. *9*, e1003715.

Tattoli, I., Sorbara, M.T., Vuckovic, D., Ling, A., Soares, F., Carneiro, L.A., Yang, C., Emili, A., Philpott, D.J., and Girardin, S.E. (2012). Amino acid starvation induced by invasive bacterial pathogens triggers an innate host defense program. Cell Host Microbe *11*, 563–575.

Velikkakath, A.K.G., Nishimura, T., Oita, E., Ishihara, N., and Mizushima, N. (2012). Mammalian Atg2 proteins are essential for autophagosome formation and important for regulation of size and distribution of lipid droplets. Mol. Biol. Cell *23*, 896–909.

Watanabe, Y., Kobayashi, T., Yamamoto, H., Hoshida, H., Akada, R., Inagaki, F., Ohsumi, Y., and Noda, N.N. (2012). Structure-based analyses reveal distinct binding sites for Atg2 and phosphoinositides in Atg18. J. Biol. Chem. *287*, 31681–31690.

Wirth, M., Joachim, J., and Tooze, S.A. (2013). Autophagosome formation—the role of ULK1 and Beclin1-PI3KC3 complexes in setting the stage. Semin. Cancer Biol. *23*, 301–309.

Ylä-Anttila, P., Vihinen, H., Jokitalo, E., and Eskelinen, E.L. (2009). 3D tomography reveals connections between the phagophore and endoplasmic reticulum. Autophagy *5*, 1180–1185.

Molecular Cell, Volume *55*

Supplemental Information

WIPI2 Links LC3 Conjugation with PI3P, Autophagosome Formation, and Pathogen Clearance by Recruiting Atg12–5-16L1

Hannah C. Dooley, Minoo Razi, Hannah E.J. Polson, Stephen E. Girardin, Michael I. Wilson, and Sharon A. Tooze

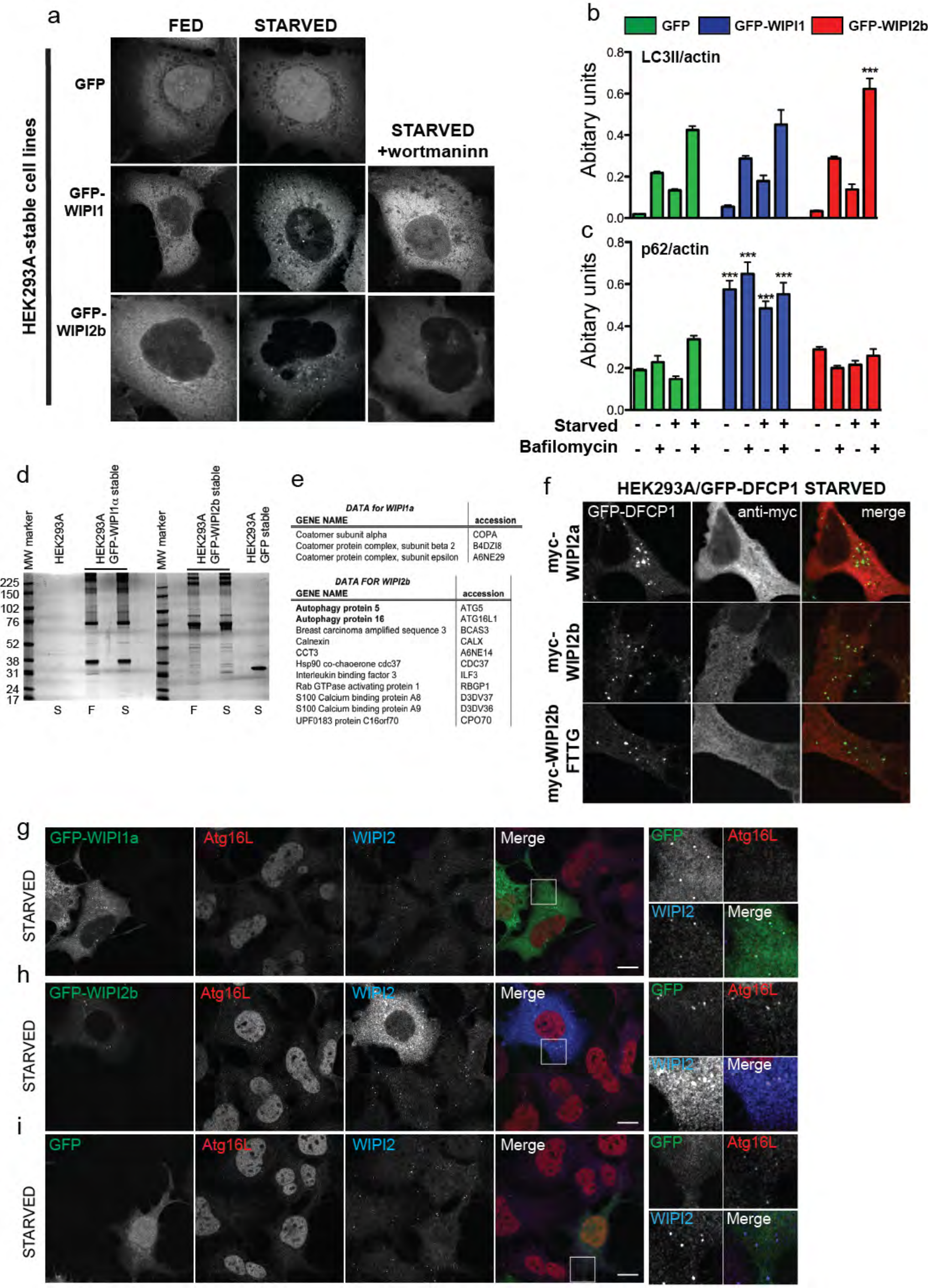

Figure S1, related to Figure 1

Figure S1, related to Figure 1.

Characterization of GFP, GFP-WIPI1 and WIPI2b stable cell lines and GFP-TRAP pulldown. ***(a)*** HEK293A stable cell lines were incubated in full medium (fed), EBSS (starved) or EBSS plus wortmannin (Starved + wortmannin) for 2 hrs then fixed and visualized by confocal microscopy. ***(b, c)*** Analysis of flux in stable cell lines. GFP, GFP-WIPI1 or GFP-WIPI2b cells (in triplicates) were incubated with full medium (Fed), full medium with BafA (Fed+Baf), EBSS (Starved) and EBSS with BafA (Starved+BafA) for 2 hrs. LC3-II and p62 signals were quantified and normalized to actin. Error SEM for three independent experiments is shown. Statistical analysis was performed by one-way anova. ***, $p<0.0005$. ***(d)*** Protein complexes were purified from stable cell lines (GFP, GFP-WIPI1 or GFP-WIPI2b) from both fed and starved conditions using GFP-Trap® subjected to SDS-PAGE and stained with colloidal stain. ***(e)*** A list of identified interacting proteins after comparison and removal of those pulled down from cells expressing GFP alone or untransfected HEK293 cells. ***(f)*** WIPI2b colocalises with DFCP-1 but WIPI2a does not. Myc-tagged WIPI2a, WIPI2b and WIPI2b FTTG were transfected into GFP-DFCP1 stable cell lines. After 24 hrs the cells were starved in EBSS for 2 hrs, fixed and labelled with anti-myc antibody. GFP-DFCP1 and myc-tagged proteins were then visualized by confocal microscopy. ***(g-i)*** Cells stably expressing (g) GFP-WIPI1a, (h) GFP-WIPI2b, or (i) GFP were starved for 2 hrs in EBSS before visualisation by confocal microscopy using antibodies against endogenous Atg16L1 and WIPI2. Scale bars are equal to 10 μm. Note: In (g) in the untransfected cell (bottom right) endogenous WIPI2 colocalizes with Atg16L1.

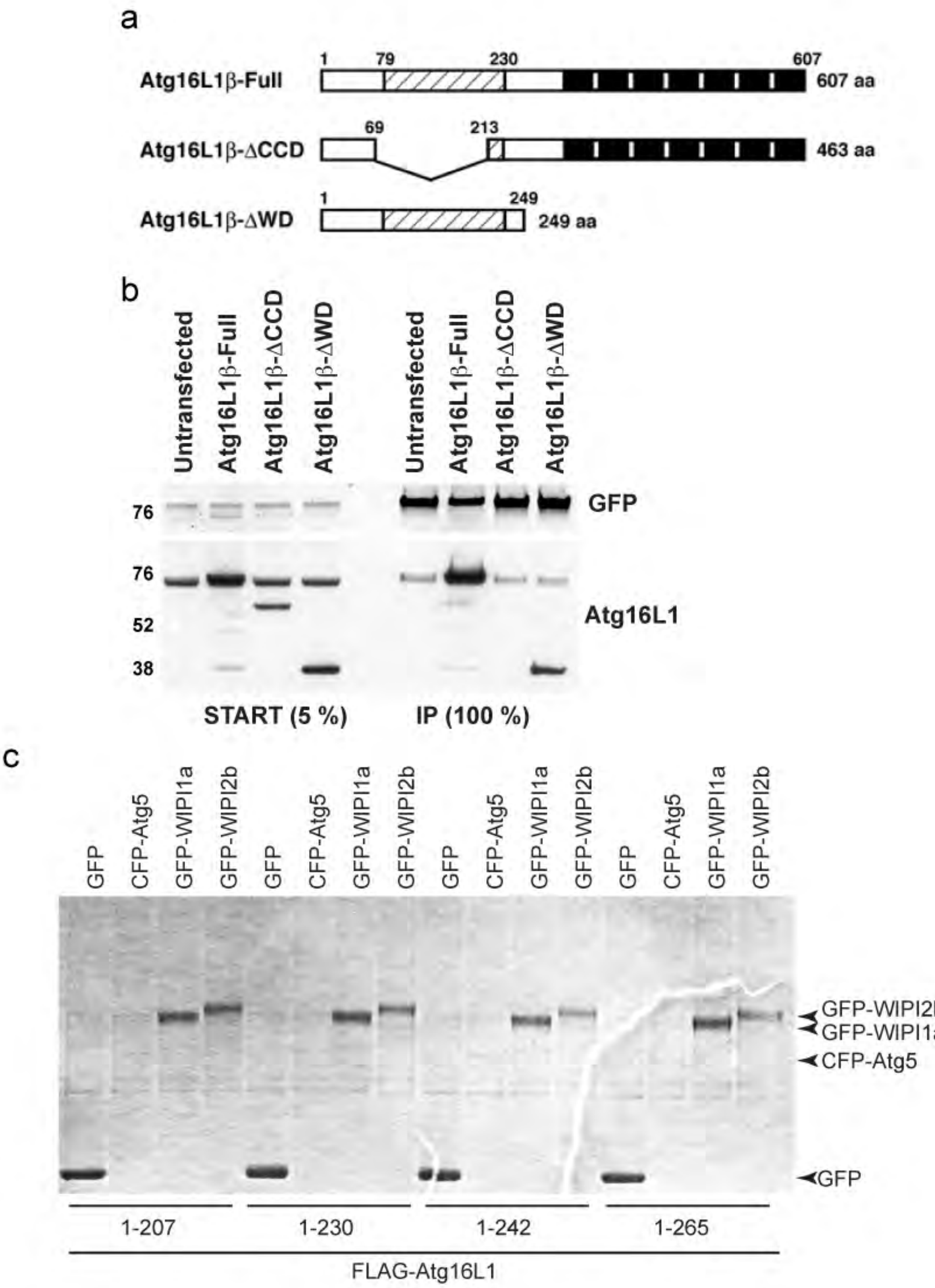

Figure S2, related to Figure 2

Figure S2, related to Figure 2.

Mapping interaction with Atg16L1. ***(a and b)*** Residues 1-249 and the coil-coil domain of human Atg16L1 are required for interaction with WIPI2b. Human Atg16L1b constructs were transfected into HEK293A cells stably expressing GFP-WIPI2b and isolated using GFP-TRAP® followed by immunoblotting with anti-Atg16 antibody. ***(c)*** Commassie blue stained gel of GFP-Trap® samples from Fig. 2c.

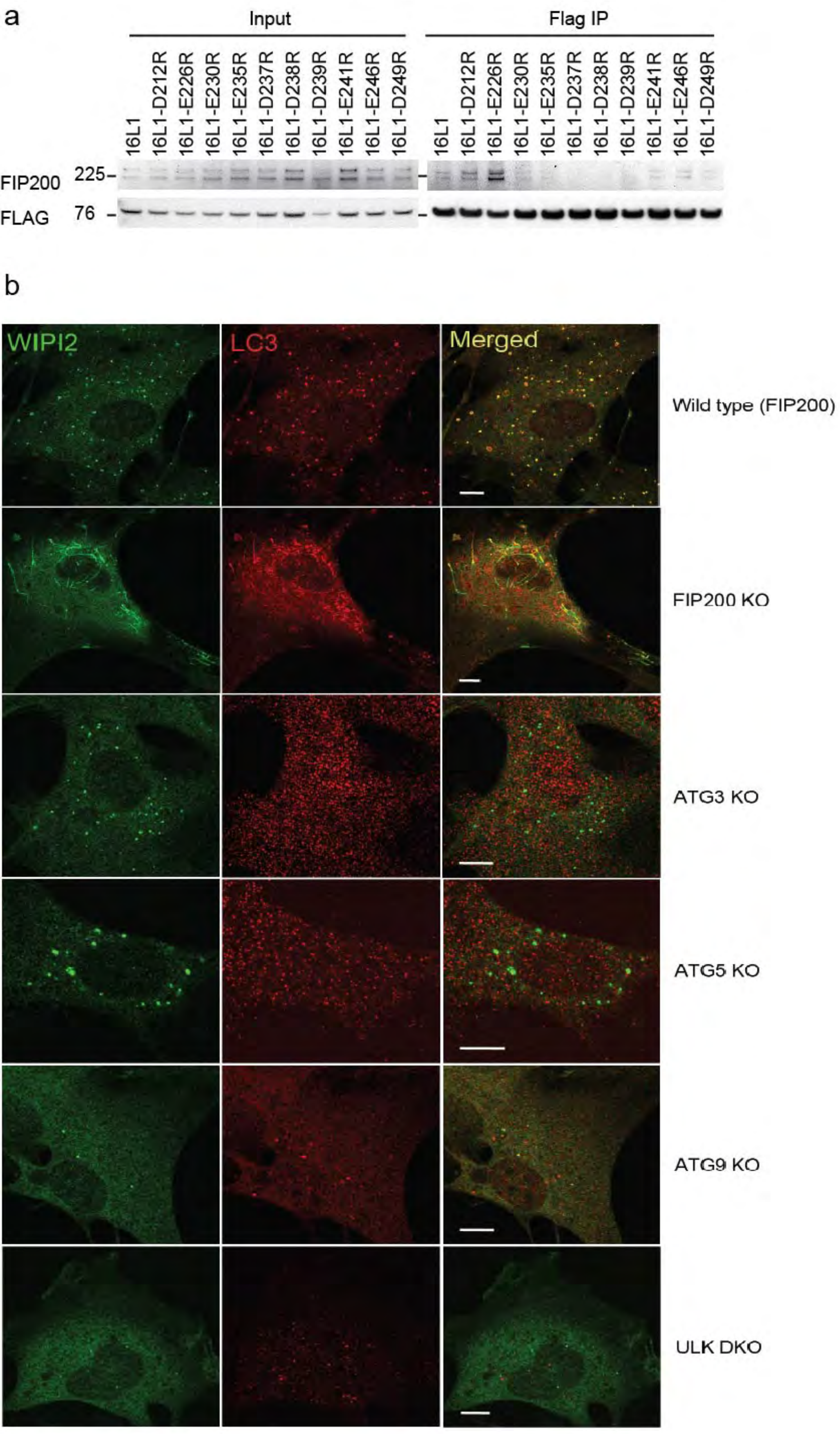

Figure S3, related to Figure 3

Figure S3, related to Figure 3. Mapping the Atg16L1 binding site in FIP200, and the localization of WIPI2 in FIP200 and other Atg-mutant MEFS. ***(a)*** Cell lysates from HEK293A cells transiently expressing GFP-WIPI2b were mixed with lysates from HEK293A cells transiently expressing FLAG-Atg16L1, or the FLAG-Atg16L1 mutants shown, and immunoprecipitated using FLAG M2 agarose beads. Tagged-protein input and bound were analysed by immunoblotting using anti-FIP200 and anti-FLAG antibodies. ***(b)*** Endogenous WIPI2 localization in FIP200, Atg3, Atg5, Atg9 and ULK1/2 Knockout MEFs. Cells were starved in EBSS for 90 mins and then fixed. WIPI2 was labelled with a monoclonal anti-WIPI2 (2A2) while LC3 was labelled with a rabbit anti-LC3 (Abcam). WIPI2 forms puncta in wild-type cells as expected. In 10% of FIP200 KO MEFs, WIPI2 is present on long tubules decorated with LC3, while the remaining 90% have undetectable WIPI2 or LC3. In Atg3 and Atg5 KO MEFS WIPI2 forms puncta that are not LC3 positive. Atg9 and ULK DKO are as previously described (McAlpine et al., 2013, Orsi et al., 2012). Atg9 KO MEFS has a small number of WIPI2-LC3-positive puncta while ULK DKO has fewer, which are not LC3-positive. Scale bars are 10μm.

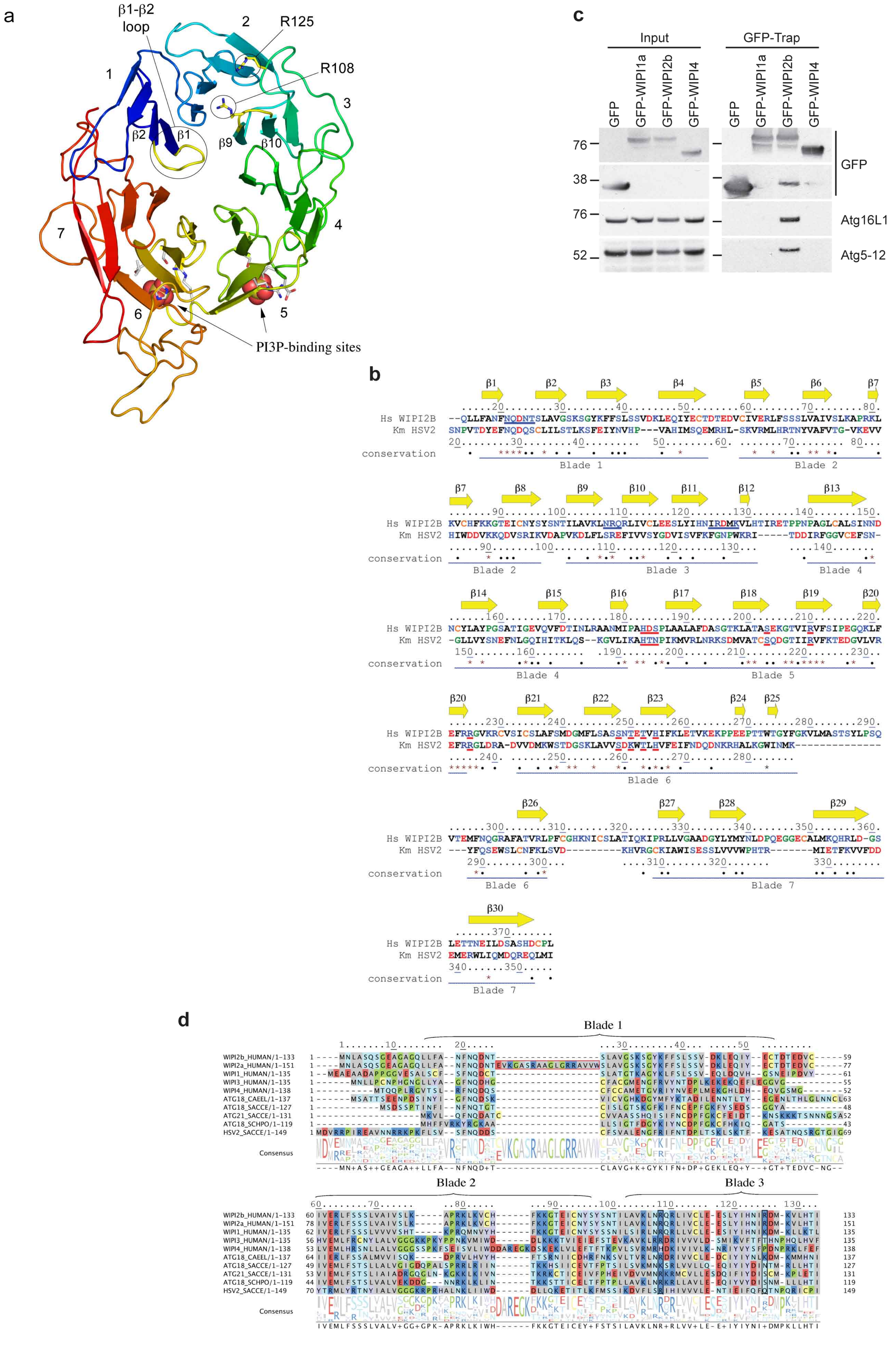

Figure S4, related to Figure 4

Figure S4, related to Figure 4. ***(a)*** A model for human WIPI2b (14-377) based on the X-ray structure of *Kluyvelomyces marxianus* Hsv2 homologue (Watanabe et al., 2012);3vu4.pdb) made with the iterative threading assembly refinement (I-TAASER;(Roy et al., 2010)). The cartoon is colored from the N-terminus with blue to red gradient. The b1-b2 and b9-b10 loops are colored yellow, R108 and R125 are shown as sticks (highlighted in the alignment with "_"). Residues that bind PI3P (highlighted in the alignment with "_") and sulfates bound in 3vu4.pdb are shown as sticks and sphere representations, respectively. ***(b)*** Structural alignment of the WIPI2b model and HSV2 (3vu4.pbd). Secondary structure in the is from 3vu4.pdb and numbering in the alignment is from Uniprot Q9Y4P8 for WIPI2 isoform 4 (WIPI2b), and J3QW34 for *Kluyvelomyces marxianus* Hsv2. Conservation symbols are '*'=identity and '•'=functional equivalence. The WIPI2b model aligned to 316 of 319 residues in 3vu4:A with an RMSD <0.89. There were 34 additional residues in loops of WIPI2b model, with half these residues (17) inserted between b25-b26 of Blade 6. ***(c)*** WIPI4 does not interact with Atg16L1. GFP-WIPI4 was pulled down from HEK293 cells transfected with GFP-WIPI2 and GFP-WIPI4 and Atg16L1 was detected by immunoblotting using anti-Atg16 and anti-GFP antibodies. ***(d)*** Comparison of WIPI2b sequence to homologues. Alignment of following sequences: human WIPI1a (Q5MNZ9 isoform 1), human WIPI2a (Q9Y4P8 isoform 1), human WIPI2b (Q9Y4P8 isoform 4), human WIPI3 (Q5MNZ6), human WIPI4 (Q9Y484), *Saccharomyces cerevisiae* ATG18 (P43601), *Schizosaccharomyces pombe* ATG18a (Q9HDZ7), *Saccharomyces cerevisiae* ATG21 (Q02887) (insertion 53-98 not shown), *Saccharomyces cerevisiae* HSV2 (P50079), *Caenorhabditis elegans* ATG18a (O16466), was generated using Jalview (Waterhouse et al., 2009). Sequences and sequence numbering are from Uniprot (Consortium, 2013). Scale numbering is from WIPI2b. Normalized consensus sequence is shown below alignment. Residues are coloured by function and strength of colouring proportional to conservation above 15% identity in each column. The boundaries of Blade 1, 2 and 3 are indicated. The 18 aa insert in WIPI2a between β1-β2 is boxed in red. R108 and R125 positions are boxed in blue.

a

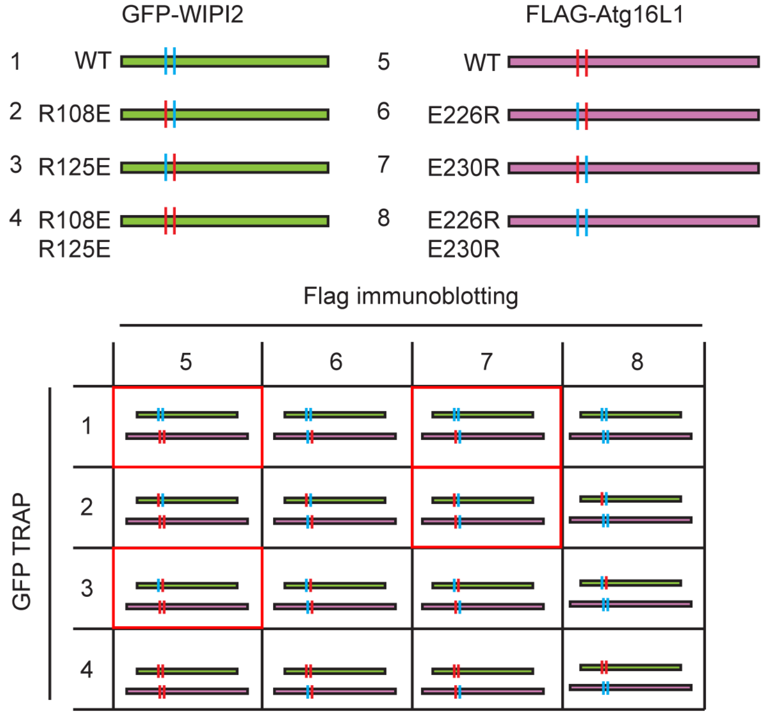

b

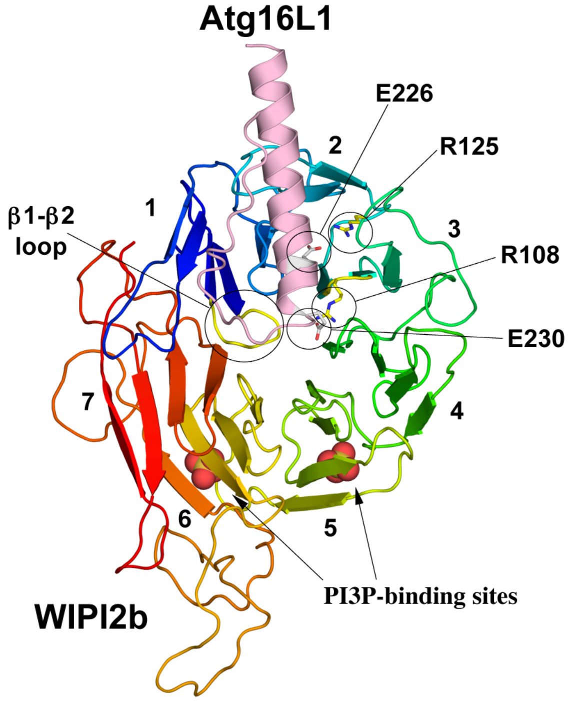

Figure S5, related to Figure 4

Figure S5, related to Figure 4. *(a)* Charge-change experiment key for Figure 4d. HEK293A cell lysates containing WIPI2b WT or point mutants no. 1-4 were mixed with HEK293A cell lysates expressing Atg16L1 WT or point mutants no. 5-8. In no. 1-4 blue vertical lines indicate the residues R108 and R125, while red vertical lines indicate the charge change mutation to E108 and E125. In no. 5-8 the red vertical lines indicate residues E226 and E230, while blue vertical lines indicate the mutation to R226 and R230. Following a GFP-TRAP® pulldown of WIPI2 the matrix indicates all the combinations tested. The boxes in red are those in which FLAG-Atg16L1 was detected after GFP-Trap® of WIPI2b (see Figure 4d). ***(b)* Model of Atg16L1 (207-246) (pink) docked onto WIPI2b (14-377) (rainbow)**. WIPI2b was modelled using 3vu4.pdb and Atg16L1 was modelled *ab initio* using I-TASSER (Roy et al., 2010). Both models were initially docked using ZDOCK for rigid-body protein-protein docking (Pierce et al., 2011), constrained by requiring E226 and E230 of Atg16L1, R108 and R125 of WIPI2b to be in the binding site. The top scoring docked model was then refined using RosettaDock (Lyskov and Gray, 2008) for local docking optimization. In the docking model shown, E230 interacts with R108.

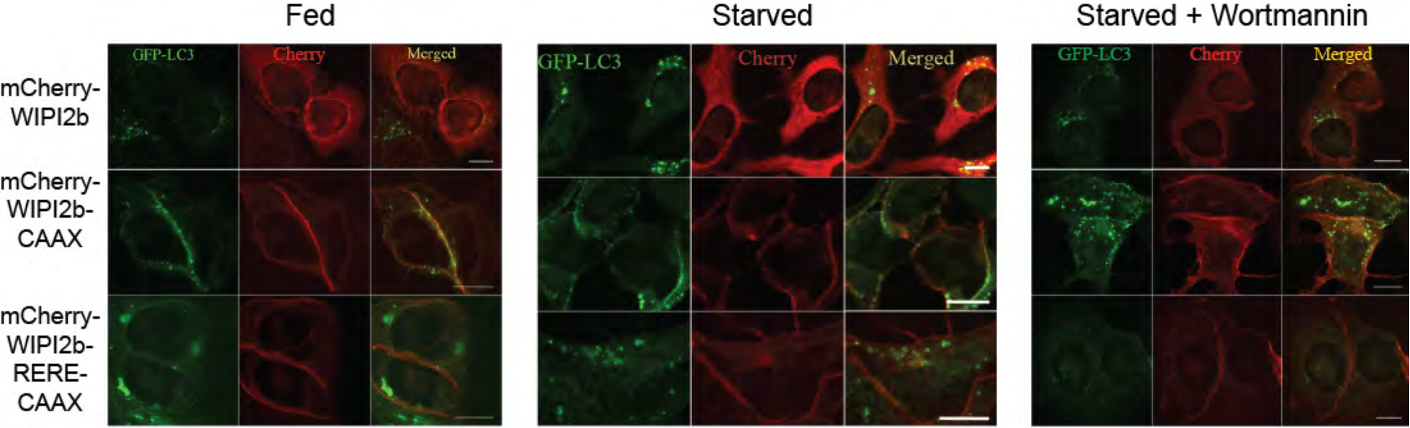

Figure S6, related to Figure 6

Figure S6, related to Figure 6. MCF7 WIPI2b-CAAX experiment. MCF7 cells transiently overexpressing the indicated mCherry constructs were either left in full medium (F), starvation medium (S) or starvation medium with wortmannin (W) for 2 hrs before being fixed and visualised by confocal microscopy. Scale bars are 10μm.

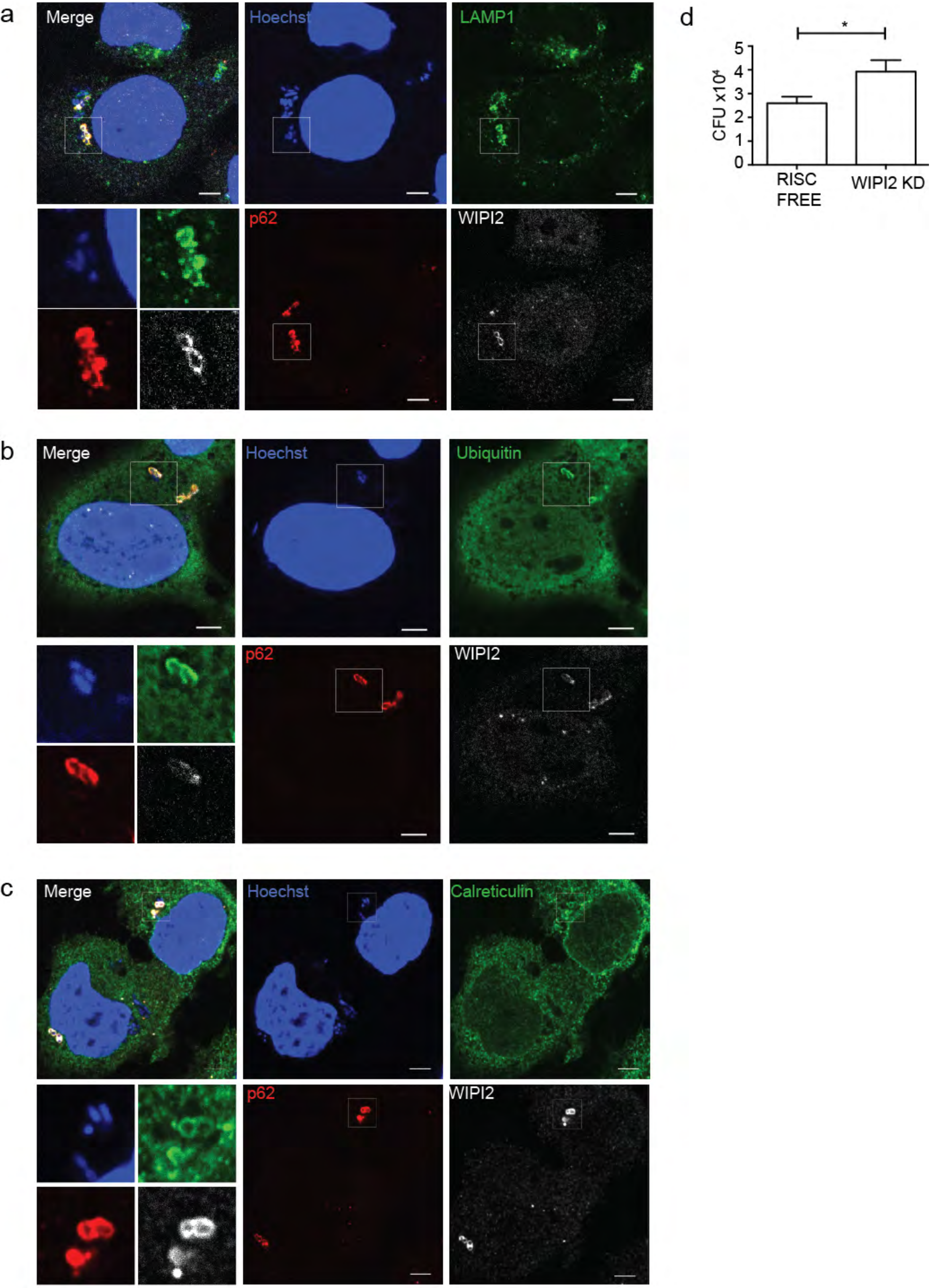

Figure S7, related to Figure 7

Figure S7, related to Figure 7. WIPI2-positive Salmonella are found in LAMP1 and UB-positive vesicles, which colocalize with ER marker calreticulin. ***(a, b, c)*** HeLa cells treated with RF siRNA for 72 hours were infected with Salmonella (MOI=100) for 1 hour. Cells were the fixed and labelled with the indicated antibodies for confocal microscopy analysis.

Loss of WIPI2 increases Salmonella proliferation. ***(d)*** HeLa cells treated with RF or WIPI2 siRNA for 72 hours were infected with Salmonella (MOI=100) for 1 hour and the colony-forming units (CFUs) determined. SEM for three independent experiments in triplicate is shown.

Movie S1, related to Figure 1. Live cell imaging of HEK293A cells stably expressing GFP-WIPI2b co-transfected with mCherry-Atg16L1. Cells were placed into EBSS at 37°C and immediately imaged using an UltraVIEW Perkin-Elmer spinning disk confocal microscope. Arrows mark the WIPI2b-positive structures shown in Fig. 1k. The arrows appear in the sequence shown in Fig. 1k, top, middle and bottom panels.

Extended Experimental Procedures:

Cell culture reagents

HeLa cells, HEK293A cells and their derivatives were grown in full medium: DMEM supplemented with 10% fetal calf serum and 4.8 mM L-glutamine. MEF cell lines were grown in full medium (FM): DMEM supplemented with 20% fetal calf serum and 4.8 mM L-glutamine. To induced autophagy, cells were washed in and incubated in Earle's balanced salt solution (EBSS) for two hours. Where indicated, cells were treated with: 100 nM bafilomycin A1 (Calbiochem), or 100 nM Wortmannin (Calbiochem). FIP200$^{-/-}$ and matched wild type immortalized MEF cell lines were a kind gift from Jun-Lin Guan (Gan et al., 2006), and Atg16$^{-/-}$ and matched immortalized MEF cell lines were kind gift from Shizuo Akira (Saitoh et al., 2008), Atg5$^{-/-}$ and matched wild type immortalized MEF cell lines were a kind gift from Noboru Mizushima (Kuma et al., 2004), Atg3$^{-/-}$ and matched wild type immortalized MEF cell lines were a kind gift from Masaaki Komatsu(Sou et al., 2008). The 293/GFP-LC3 cells are as previously described (Chan et al., 2007). HEK293 cell lines stably expressing GFP, GFP-WIPI1a and GFP-WIPI2b were established by transient transfection of the pEGFP-constructs, selection with G418 (800 μg/mL) and cloned by limiting dilution and then selected for low level expression by FACs sorting before being maintained in the presence of G418 at 400μg/mL. GFP-LC3 MEFs were kindly provided by Fiona McAlpine (LRI, CRUK). HEK239A/DFCP1 (201 cells) cells were provided by N. T. Ktistakis (Babraham Institute, Cambridge, UK).

Lipofectamine 2000 (Invitrogen) was used according to the manufacturer's instructions for transient transfection of HeLa, HEK293A and derivative cells. DNA plasmids were used at a concentration of 1μg/mL of transfection mix. For RNA interference of HeLa, HEK293A and derivate cells, cells were transfected with the relevant siRNA oligo using Oligofectamine (Invitrogen), followed by a second transfection using Lipofectamine 2000 (Invitrogen) the following day, both used according to the manufacturer's instructions. Final concentration of siRNA oligos was 50 nM. siRNA Oligos used: Darmacon: D-001220-01 (RISC-Free, control), L-021033-01 (Atg16L1), LU-021117-00 (RB1CC1/FIP200), Qiagen: Hs_WIPI2_4 (WIPI2). Knock-down and rescue experiments were performed by using oligofectamine with siRNA on day one, followed by lipofectamine with siRNA and DNA on day two. Rescue assay was performed 48 hours after lipofectamine treatment. For transfecton of MEF cells, jetPRIME (Polyplus-

transfection) was used according to the manufacture's instructions. DNA plasmids were used at a concentration of 1µg/mL of transfection mix. MEF rescue assays were performed 24 hours after transient transfection with jetPRIME.

pEGFP-C1-WIPI1a construct (Accession No. AY691424) was a gift from Tassula Proikas-Cezanne (University of Tuebingen, Germany) and mutated to agree with gene bank sequence BC039867 (Val to Ala at amino acid position 81). GFP-WIPI4 was a gift from Noboru Mizushima (University of Tokyo). GFP-LC3, FLAG-Atg16L1 WT, 1-265 and 79-623 constructs were a gift from Tamotsu Yoshimori (Osaka Univeristy). FLAG-Atg16L2 construct was a gift from Mitsunori Fukuda (Tohoku University). pEGFP-WIPI2a and WIPI2b were generated by PCR amplification, respectively. The lipid-binding mutant (FRRG to FTTG) and point mutants of GFP-WIPI2b, and point mutants and truncations of FLAG-Atg16L1 were generated using QuikChange Site-directed Mutagenesis Kit (Stratagene). WIPI2-CAAX constructs were generated using PCR amplification and site-directed mutagenesis. Primers, see Table S1.

Mouse monoclonal antibodies: anti-LC3 (Nanotools, 0231-100/LC-3-5F10), anti-myc 9E10 (CRUK), anti-Atg16L1 (MBL, M150-3B), anti-FLAG-M2 (Sigma, F3165), anti-GFP (CRUK, 3E1), anti-HA (Covance, MMS-101R), anti-LAMP1 (BD Biosciences, 555801) anti-p62 (BD Biosciences, 610833), anti-ubiquitin (MBL, D058-3), and anti-WIPI2 (polson et al.). Rabbit polyclonal anti-Atg16L (Cosmo Bio, TMD-PH-AT16), anti-FLAG (Sigma, F7425), anti-LC3 (Abcam, ab48394), anti-β actin (Abcam, ab8227), anti-Atg5 (Cosmobio, TMD-PH-At5), anti-Atg12 (Cell signalling, #2010), anti-FIP200 (Bethyl Laboratories, A301-536A), anti-RFP (MBL, PM005), anti-WIPI1(Polson et al., 2010) and anti-WIPI2(Polson et al., 2010). Guinea pig polyclonal anti-p62 (Progen, cat. No. GP62-C). Chicken polyclonal anti-calreticulin (ab2908). Antibodies were used at manufacturers suggested concentrations. Secondary antibodies used for IF were anti-rabbit IgG Alexa Fluor 488, and 555, anti-mouse IgG Alexa Flour 488, 555, and 647, and anti-guinea pig IgG Alexa Fluor 555. HRP-conjugated secondary antibodies used for WB were from GE Healthcare.

Protein complex purification and Mass Spectroscopy

Fed or starved cell pellets washed in PBS were lysed in 10 mM Tris-HCl pH7.5, 150 mM NaCl, 0.5 mM EDTA, 0.5 % NP40, 2x Complete protease inhibitor (Roche,), 1x PhosSTOP (Roche) and the lysate clarified by high speed centrifugation (20,000xg 10 min). Lysates

were diluted to 0.2% NP-40 and incubated with GFP-Trap® beads at 4°C for 1 hour. Pelleted beads were washed 3 times with 10 mM Tris-HCl pH7.5, 150 mM NaCl, 0.5 mM EDTA, 1 % NP40, 1x Complete protease inhibitor, 1x PhosSTOP and protein complexes eluted with 2x Laemmli sample buffer at 65°C for 10 min.

Immunoprecipitation:

Endogenous immunoprecipiation: cells were permeabilised using TNTE buffer (1% triton TX-100, 20 mM Tris HCL, pH7.5, 150 mM NaCl, 5 mM EDTA, 1 X Complete protease inhibitor) and the lysates used for immunoprecipitation with monoclonal anti-WIPI2 (A2A) overnight before washing with TNTE. Crosslinker was cleaved by boiling the sample in Laemmli sample buffer containing 50 mM DTT and boiled for 10 minutes before resolving by SDS-PAGE (4-12% BisTris NUPAGE gels, Invitrogen). GFP-tagged proteins were immunoprecipitated using GFP-Trap® beads as for mass spectrometry, but with 500 mM NaCl in the wash buffer and no PhosSTOP in any buffer. FLAG-tagged proteins were immunoprecipitated using anti-FLAG M2 affinity gel (Sigma) for one hour with the same lysis and wash buffers as used for GFP-Trap®.

In vitro translation

For binding assays using *in vitro* translated proteins, cells overexpressing GFP-tagged proteins were used for GFP- Trap®as described as above. The GFP-Trap® product was then mixed with in vitro translated FLAG-tagged protein. Mixtures were incubated for one hour before washing with GFP-Trap® wash buffer. The product was run on SDS-PAGE (4-12% BisTris NUPAGE gels, Invitrogen) before Coomasssie Blue staining, drying and auto-radiation. *In vitro* translated protein was produced using TNT® Quick Coupled Transcription/Translation System (Promega) and EasyTag™ L-[^{35}S]-methonine (PerkinElmer).

Confocal microscopy

Cells were grown on coverslips, fixed with 3% paraformaldehyde for 20 minutes before permeablisation with either 50 ug/mL digitonin (endogenous Atg16 staining), or with room temperature methanol (endogenous LC3 staining) for five minutes. Coverslips were blocked in 5% BSA (Roche) for one hour, incubated with primary antibody in 1% BSA for one hour, washed in 1% BSA, incubated with secondary antibody in 1% BSA for

one hour, before final washing with PBS and water. LC3 puncta formation was assessed by Imaris image analysis software. Salmonella experiments were quantified using Imaris-assisted manual counting.

Correlative Light and Electron Microscopy (CLEM)

CLEM was performed as previously described (Razi and Tooze, 2009). In Fig. 1d-e, Atg16L1 MEFs were fixed with PFA 2% + Glut 2% in PBS, followed by Digitonin permilization for 5 mins (50ng/ul), then washed twice in 10% BSA for 2x5min. Cells were stained with anti-WIPI2 antibody, and secondary as described above. HEK293 cells expressing GFP-WIPI2b RERE (Fig. 4i, j), or MCF7 cells expressing mCherry-WIPI2b-CAAX and GFP-LC3 (Fig. 6e) were processed and imaged as previously described (Orsi et al., 2012).

Live cell imaging

Live cell imaging of HEK293 cells stably expressing GFP-WIPI2b co-transfected with mCherry-Atg16L1. Cells were placed into EBSS at 37°C and immediately imaged using an UltraVIEW Perkin-Elmer spinning disk confocal microscope. Images were processed using ImageJ and MetaMorph software.

Modeling

A model for human WIPI2b (14-377) was made using the X-ray structure of the *Kluyvelomyces marxianus* Hsv2 homologue ((Watanabe et al., 2012); 3vu4.pdb) with the iterative threading assembly refinement (I-TASSER) (Roy et al., 2010). The model geometry was refined using COOT (Emsley et al., 2010). Final backbone geometries were 100% in allowed and preferred regions of the Ramachandran plot, all sidechain geometries were acceptable and no clashes were detected. Structural alignment of the WIPI2b model with 3vu4:A using TMalign (Zhang and Skolnick, 2005) revealed that the model aligned to 316 of 319 residues in 3vu4:A, with an RMSD <0.89. An ab initio model for human Atg16L1 (207-246) was using I-TASSER and model geometry refined using COOT.

Statistical analysis

Performed using Prism 6 software as detailed in the figure legends.

Primers used in this study

Primer	Sequence
WIPI2b siRES-SDM-1	GTGGGTGCCGCCGACGGGTATCTTTACATGTACAAC
WIPI2b siRES-SDM-2	GTGGGTGCCGCCGATGGTTATCTTTACATGTACAAC
HA-WIPI2CAAX-forward	ATGAATTCACCATGTACCCATACGATGTTCCAGATTACGCT AACCTGGCGAGCCAGAGCGGG
Reverse-WIPI2CAAX	AACTTAAGCTTTCACATAATTACAC
mCherry-WIPI2CAAX-forward	ATCTCGAGGGAACCTGGCGAGCCAGAGCGGG
Atg16L1 mus 1-230 SDM	GAAGCAGCAAAGGAATAGCCTCTACCTGTTGAAC
Atg16L1 mus 1-242 SDM	GATGACATTGAAGTCTAGATTGTGGATGAGACC
Atg16L1 mus 1-207 SDM	CAATCGCCTCAATGCATAGGAGAATGAGAAGGAC
WIPI2b-R108E	GCTGTGAAGCTCAACgagCAGAGGCTGATAGTATG
WIPI2b-R125E	CATCCACAACATTgagGACATGAAGGTGCTGC
Atg16L1 mus E208R	CCAATCGCCTCAATGCAAGGAATGAGAAGGACTCC
Atg16L1 mus D212R	GCAGAGAATGAGAAGcgcTCCAGGAGGCGTCAAGC

Atg16L1 mus E226R	CTGCAGAAGGAGCTTGCAagaGCAGCAAAGGAACCTCTAC
Atg16L1 mus E230R	GAGCTTGCAGAAGCAGCAAAGagaCCTCTACCTGTTGAAC
Atg16L1 mus E235R	CAAAGGAACCTCTACCTGTTagaCAGGATGATGACATTGAAG
Atg16L1 mus D237R	CCTCTACCTGTTGAACAGcgtGATGACATTGAAGTCATTG
Atg16L1 mus D238R	CTACCTGTTGAACAGGATcgtGACATTGAAGTCATTGTGG
Atg16L1 mus D239R	CCTGTTGAACAGGATGATcgcATTGAAGTCATTGTGGATG
Atg16L1 mus E241R	GAACAGGATGATGACATTcgtGTCATTGTGGATGAGACC
dCAAX SDM	CCATGATTCTTCGGACTGACtgaAGAAAAGATGGTAAAAAGAAG
Atg16L1 mus E226R E230R	GAGCTTGCAagaGCAGCAAAGagaCCTCTACCTGTTGAAC
Atg16L1 mus D237R D239R	CCTGTTGAACAGcgtGATcgcATTGAAGTCATTGTGGATG
Atg16L1 mus E246R	CATTGAAGTCATTGTGGATaggACCTCAGACCACACAGAAG
Atg16L1 mus D249R	CATTGTGGATGAGACCTCAcgcCACACAGAAGAGACCTCTC
Atg16L1 mus E253R	GACCTCAGACCACACAGAAcggACCTCTCCCGTCCGAGCTG
WIPI2 mut FTTG	GGACAAAAACTCTTTGAGTTTACGACAGGAGTAAAGAGGTGCGTG

WIPI1 dCT	ACAGCTTGCTTGGCTCAtaaACAACAGAAGAGAATAAAG
WIPI2 dCT	CACCGGCTGGACGGCAGTtaaGAAACGACCAATGAGATC

References

CONSORTIUM, U. 2013. Update on activities at the Universal Protein Resource (UniProt) in 2013. *Nucleic Acids Res.*

EMSLEY, P., LOHKAMP, B., SCOTT, W. G. & COWTAN, K. 2010. Features and development of Coot. *Acta Crystallogr D Biol Crystallogr.* International Union of Crystallography.

GAN, B., PENG, X., NAGY, T., ALCARAZ, A., GU, H. & GUAN, J.-L. 2006. Role of FIP200 in cardiac and liver development and its regulation of TNFα and TSC–mTOR signaling pathways. *The Journal of Cell Biology,* 175**,** 121-133.

KUMA, A., HATANO, M., MATSUI, M., YAMAMOTO, A., NAKAYA, H., YOSHIMORI, T., OHSUMI, Y., TOKUHISA, T. & MIZUSHIMA, N. 2004. The role of autophagy during the early neonatal starvation period. *Nature,* 432**,** 1032-6.

LYSKOV, S. & GRAY, J. J. 2008. The RosettaDock server for local protein-protein docking. *Nucleic Acids Res.*

MCALPINE, F., WILLIAMSON, L., TOOZE, S. A. & CHAN, E. Y. W. 2013. Regulation of nutrient-sensitive autophagy by uncoordinated-51 like kinases 1 and 2. *Autophagy,* 9**,** 361-373.

ORSI, A., RAZI, M., DOOLEY, H., ROBINSON, D., WESTON, A., COLLINSON, L. & TOOZE, S. 2012. Dynamic and transient interactions of Atg9 with autophagosomes, but not membrane integration, is required for autophagy. *Mol Biol Cell,* 23**,** 1860-1873.

PIERCE, B. G., HOURAI, Y. & WENG, Z. 2011. Accelerating protein docking in ZDOCK using an advanced 3D convolution library. *PLoS ONE.*

RAZI, M. & TOOZE, S. A. 2009. Correlative light and electron microscopy. *Methods Enzymol,* 452**,** 261-75.

ROY, A., KUCUKURAL, A. & ZHANG, Y. 2010. I-TASSER: a unified platform for automated protein structure and function prediction. *Nature protocols.*

SOU, Y.-S., WAGURI, S., IWATA, J.-I., UENO, T., FUJIMURA, T., HARA, T., SAWADA, N., YAMADA, A., MIZUSHIMA, N., UCHIYAMA, et al. 2008. The Atg8 Conjugation System Is Indispensable for Proper Development of Autophagic Isolation Membranes in Mice. *Mol. Biol. Cell,* 19**,** 4762-4775.

WATERHOUSE, A. M., PROCTER, J. B., MARTIN, D. M., CLAMP, M. & BARTON, G. J. 2009. Jalview Version 2--a multiple sequence alignment editor and analysis workbench. *Bioinformatics,* 25**,** 1189-91.

ZHANG, Y. & SKOLNICK, J. 2005. TM-align: a protein structure alignment algorithm based on the TM-score. *Nucleic Acids Res.*
